# Modulation of cellular signaling by herpesvirus-encoded G protein-coupled receptors

**DOI:** 10.3389/fphar.2015.00040

**Published:** 2015-03-09

**Authors:** Sabrina M. de Munnik, Martine J. Smit, Rob Leurs, Henry F. Vischer

**Affiliations:** Amsterdam Institute for Molecules Medicines and Systems – Division of Medicinal Chemistry, Department of Chemistry and Pharmaceutical Sciences, VU University Amsterdam, AmsterdamNetherlands

**Keywords:** human herpesvirus, viral GPCR, KSHV, HCMV, EBV, chemokine, chemokine receptor, review

## Abstract

Human herpesviruses (HHVs) are widespread infectious pathogens that have been associated with proliferative and inflammatory diseases. During viral evolution, HHVs have pirated genes encoding viral G protein-coupled receptors (vGPCRs), which are expressed on infected host cells. These vGPCRs show highest homology to human chemokine receptors, which play a key role in the immune system. Importantly, vGPCRs have acquired unique properties such as constitutive activity and the ability to bind a broad range of human chemokines. This allows vGPCRs to hijack human proteins and modulate cellular signaling for the benefit of the virus, ultimately resulting in immune evasion and viral dissemination to establish a widespread and lifelong infection. Knowledge on the mechanisms by which herpesviruses reprogram cellular signaling might provide insight in the contribution of vGPCRs to viral survival and herpesvirus-associated pathologies.

## INTRODUCTION

### G PROTEIN-COUPLED RECEPTORS

**FIGURE 1:**
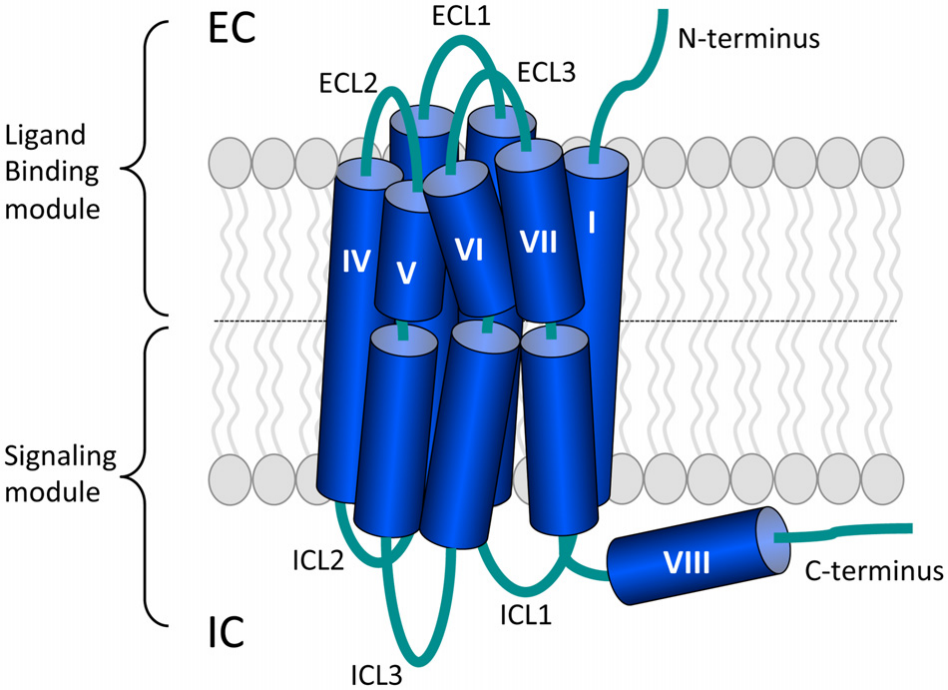
**The general architecture of class A G protein-coupled receptors (GPCRs).** Shown are the three extracellular loops (ECL1-3) and the N-terminus in the EC region and the three intracellular loops (ICL1-3) and the C-terminus in the IC region. The seven transmembrane (TM) helices are arranged in a counter-clockwise manner and contain a number of proline-dependent kinks that divide the GPCR into the ligand binding module and the module that binds downstream effectors such as G proteins. The C-terminus of many GPCRs is folded into an eighth helix that runs parallel to the plasma membrane and is often anchored to the membrane via a palmitoylation site. Image is based on (Katritch et al., 2012).

G protein-coupled receptors (GPCRs) form the largest family of transmembrane receptors (Pierce et al., 2002). GPCRs are composed of seven transmembrane helices (TMs) surrounding a central cleft that are connected by three intracellular and three extracellular loops (ICLs and ECLs, respectively). The amino terminus (N-terminus) and carboxyl terminus (C-terminus) are located at the extracellular and intracellular site, respectively (Katritch et al., 2012; **Figure 1**). The human genome encodes more than 800 GPCRs (Fredriksson et al., 2003) and this amount reflects the large diversity of extracellular ligands that they bind. GPCRs respond to ligands ranging from light, odorants, ions, and catecholamines to neuropeptides and large glycoprotein hormones (Granier and Kobilka, 2012). GPCRs are involved in nearly all physiological processes, but also in many pathological conditions and 30–40% of the current drugs on the market target GPCRs (Wise et al., 2002). Recent advances in protein engineering and crystallography aided in the exponential growth of available GPCR crystal structures (Tautermann, 2014). These structures provide insight in GPCR activation and will aid in drug discovery processes (Katritch et al., 2013).

**FIGURE 2:**
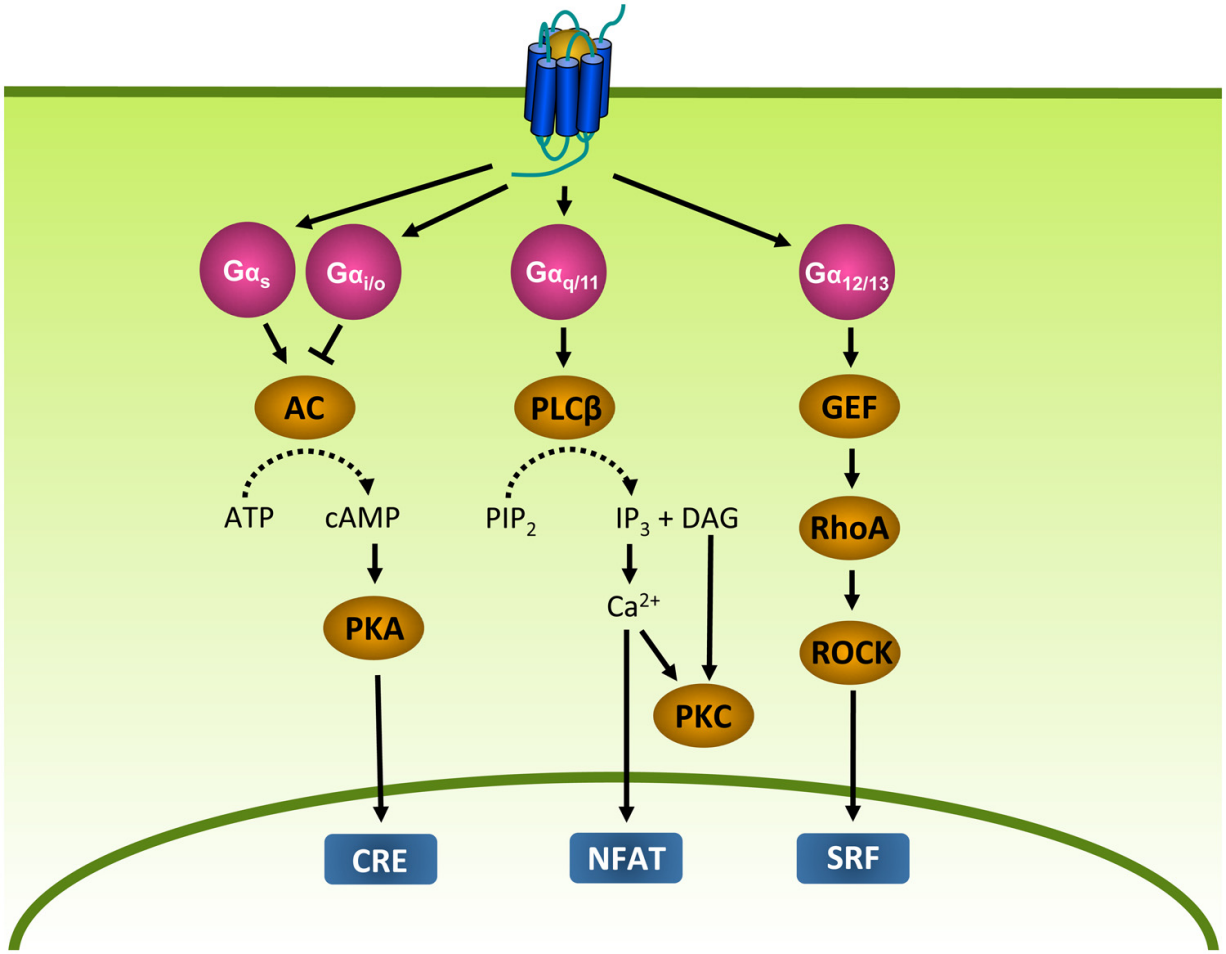
**G protein-dependent signaling.** Gα proteins are divided into Gα_s_, Gα_i_, Gα_q_, and Gα_12/13_ protein families that regulate different effector proteins such as AC and PLC. Effector proteins produce second messengers (e.g., cAMP) that subsequently activate transcription factors such as CRE, NFAT and SRF. AC, adenylyl cyclase; ATP, adenosine triphosphate; cAMP, cyclic adenosine monophosphate; CRE, cAMP-responsive element; DAG, diacylglycerol; GEF, guanine nucleotide exchange factor; IP_3_, inositol 1,4,5-triphosphate; NFAT, nuclear factor of activated T-cells; PIP_2_, phosphatidylinositol-4,5-bisphosphate; PKA, protein kinase A; PKC, protein kinase C; PLCβ, phospholipase Cβ; RhoA, Ras homolog gene family A; ROCK, RhoA kinase; SRF, serum response factor.

The main function of GPCRs is to enable cells to respond to their environment by converting extracellular stimuli into intracellular responses. Upon binding of a stimulating ligand (i.e., agonist), GPCRs undergo conformational changes that lead to the activation of heterotrimeric GTP binding proteins (G proteins; Oldham and Hamm, 2008). G proteins are composed of a α, β, and γ subunit. In its inactive state, Gα is bound to GDP. However, when activated by a GPCR, GDP is exchanged for GTP (Oldham and Hamm, 2007). As a consequence, the G protein dissociates from the GPCR and the Gα and Gβγ subunits activate effector proteins that produce second messengers, leading to the activation of transcription factors and eventually cellular responses (Oldham and Hamm, 2008). Gα proteins can be subdivided into four families (**Figure 2**). Gα_s_ proteins stimulate adenylyl cyclase (AC) and the subsequent production of cyclic AMP (cAMP), whereas Gα_i/o_ proteins inhibit AC. cAMP in turn activates protein kinase A (PKA), leading to the activation of cAMP-responsive element (CRE). Gα_q/11_ proteins stimulate phospholipase Cβ (PLCβ), an enzyme that catalyzes the formation of inositol 1,4,5-triphosphate (IP_3_) and diacylglycerol (DAG) from phosphatidylinositol-4,5-bisphosphate (PIP_2_). IP_3_ in turn increases intracellular Ca^2+^ levels by activation of the IP_3_ receptor on the endoplasmic reticulum (ER), resulting in the subsequent activation of protein kinase C (PKC) and nuclear factor of activated T-cells (NFAT). DAG activates PKC (Rohini et al., 2010). Gα_12/13_ proteins activate the small G protein RhoA through the activation of guanine nucleotide exchange factors (GEFs; McCudden et al., 2005). RhoA in turn activates RhoA kinase (ROCK) and subsequently serum response factor (SRF), which regulates a variety of cellular responses such as cytoskeletal rearrangement and cell proliferation (Heng and Koh, 2010). In addition to Gα subunits, the Gβγ subunits themselves are known to regulate the activity of PLCβ, several AC isoforms and different ion channels (McCudden et al., 2005; Milligan and Kostenis, 2006). Furthermore, GPCRs are able to activate signaling in a G protein-independent manner, for example via β-arrestins (see “Desensitization and Intracellular Receptor Trafficking of Viral GPCRs;” Tilley, 2011).

### THE CHEMOKINE RECEPTOR SYSTEM

**FIGURE 3:**
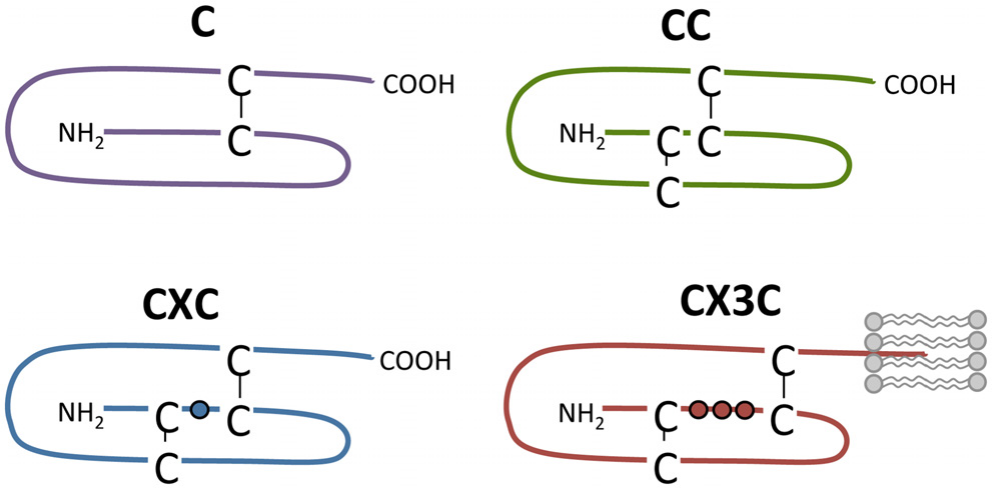
**Chemokine subclasses.** Chemokines are divided into four families according to the number and spatial organization of conserved cysteine residues in their N-terminus. Disulfide bridges are shown as black lines. The transmembrane domain of CX3CL1 is depicted by lipids (in gray).

Chemokines bind to chemokine receptors, which form a subfamily of GPCRs. Chemokines are secreted proteins (7–12 kDa) that play a key role in the immune system as they coordinate the migration of leukocytes during inflammation and immune surveillance (Rossi and Zlotnik, 2000; Charo and Ransohoff, 2006). So far, 43 chemokines have been identified in human and they are divided into four families: C, CC, CXC, and CX3C (**Figure 3**). Their classification is based on the number and arrangement of conserved cysteine residues in the N-terminus of chemokines that form disulfide bonds to stabilize tertiary folding. In the CC, CXC, and CX3C subfamily, none, a single or three amino acids are inserted between the first two of the four conserved cysteine residues, respectively (**Figure 3**). In the C subfamily of chemokines, the first and third cysteine residues are lacking and only one disulfide bond is present (Zlotnik et al., 2006; Blanchet et al., 2012; **Figure 3**). Alternatively, chemokines are divided according to their expression and function. The expression of inflammatory chemokines is induced under inflammatory conditions while homeostatic chemokines are constitutively expressed and are involved in physiological processes such as homeostatic leukocyte homing (Blanchet et al., 2012). Chemokines bind to glycosaminoglycans (GAGs) on endothelial cells and the extracellular matrix to immobilize into a chemotactic gradient to direct migrating cells (Salanga and Handel, 2011; Mortier et al., 2012). The importance of GAG binding has been demonstrated by chemokine mutants that are deficient in GAG binding and unable to recruit cells *in vivo* (Hamel et al., 2009). Additionally, CX3CL1 and CXCL16 are membrane-tethered and facilitate cell–cell adhesion with cells expressing their respective cognate receptors CX3CR1 and CXCR6, respectively (Ludwig and Weber, 2007).

**FIGURE 4:**
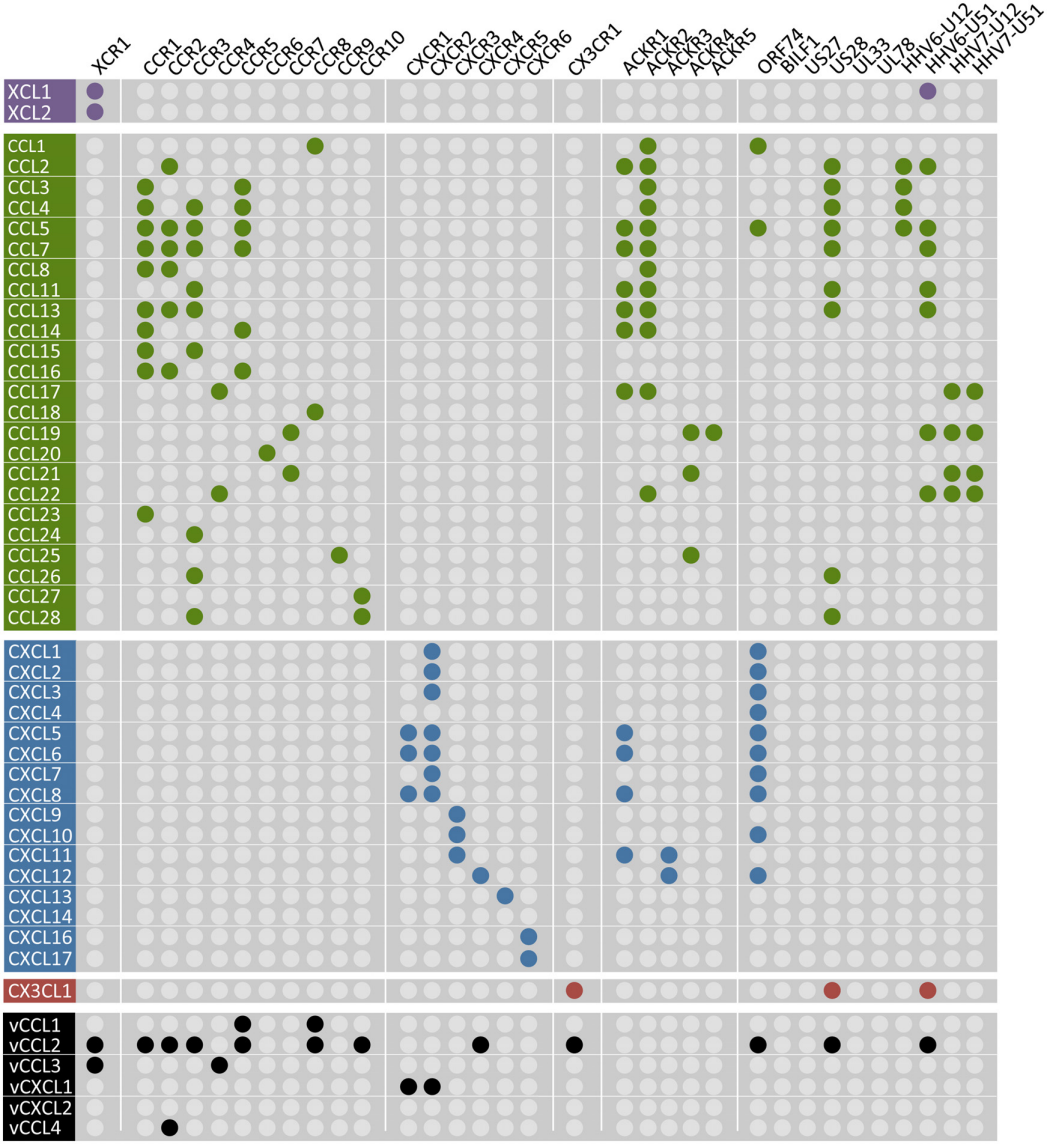
**Chemokines and their human and viral receptors.** The chemokines (vertical) are divided into four families (colors match with **Figure 3**) and the virus-encoded chemokines are also included at the bottom in black. Human chemokine receptors (horizontal) are classified according to the chemokines they bind and the a-typical chemokine receptors-5 (ACKR1-5) are also included. Viral receptors are depicted on the right. A colored dot represents the pairing of a chemokine to a specific receptor. One receptor can bind multiple chemokines and vice versa. No receptor has hitherto been identified for CXCL14 and the vGPCRs BILF1, US27, UL33, and UL78 are classified as orphan receptors as no chemokines have been identified to bind these receptors. The distribution of the colored dots shows that human chemokine receptors only bind chemokines within their own class. However, ACKR1 and some vGPCRs cross this boundary as they bind CC, CXC, and CX3CL1 chemokines. Moreover, KSHV-encoded vCCL2 binds promiscuously to XC, CC, CXC, and CX3C chemokine receptors. The diagram is based on (Bachelerie et al., 2014; Steen et al., 2014).

To date, 23 chemokine receptors have been identified, which are classified according to the specific subclass of chemokines that they bind. Only one XC and one CX3C receptor exist, whereas ten CC and six CXC receptors have been identified as of yet. The chemokine/chemokine receptor system is rather complex as many receptors can bind multiple chemokines and vice versa (**Figure 4**). Activated chemokine receptors mainly signal through Gα_i/o_ proteins to mediate chemotaxis (Neptune and Bourne, 1997). Via Gβγ subunits, chemokine receptors activate PI3Kγ and PLCβ, the latter resulting in an increased Ca^2+^ flux (Thelen, 2001). Furthermore, chemokine receptors activate mitogen-activated protein (MAP) kinases such as extracellular-signal-regulated kinases ERK1/2, p38 and c-Jun N-terminal kinases (JNK) but also Rho GTPases (e.g., RhoA and Rac) via Gα_12/13_ that mediate the reorganization of the actin cytoskeleton (Thelen, 2001; Thelen and Stein, 2008; Cotton and Claing, 2009). Besides the classical chemokine receptors, five atypical chemokine receptors (ACKR) have been identified, named ACKR1 (DARC), ACKR2 (D6), ACKR3 (CXCR7), ACKR4 (CCX-CKR), and ACKR5 (CCRL2; **Figure 4**). These receptors do not induce migration upon chemokine binding or activate G protein-dependent signaling, but recruit β-arrestin (Galliera et al., 2004; Rajagopal et al., 2010; Ulvmar et al., 2011; Canals et al., 2012; Watts et al., 2013). The ACKRs are believed to acts as decoy receptors that scavenge chemokines from the extracellular environment to limit the recruitment of leukocytes (Bonecchi et al., 2010). However, it was recently proposed that G_i/o_ proteins impair ACKR4-mediated signaling. Preventing the interaction with G_i/o_ proteins by treating cells with pertussis toxin (PTX) unmasked signaling of ACKR4 to CRE (Watts et al., 2013). Furthermore, ACKR2 activates a β-arrestin1-dependent signaling cascade, resulting in the phosphorylation of cofilin (Borroni et al., 2013).

Besides their role in the immune system, chemokine receptors are also involved in other physiological processes including development, tissue repair, angiogenesis, and neuroprotection (Strohmann et al., 1974; Kiefer and Siekmann, 2011; Jaerve and Muller, 2012; Martins-Green et al., 2013). Dysregulation of chemokines and their receptors may result in an excessive infiltration of leukocytes into tissue. Indeed, chemokine receptors are involved in several inflammatory diseases such as arthritis, multiple sclerosis, asthma, psoriasis, Crohn’s disease and atherosclerosis (Bendall, 2005; Cardona et al., 2013; Marra and Tacke, 2014). Moreover, chemokine receptors also play a role in oncogenesis by inducing proliferation and metastasis (Koizumi et al., 2007; Wu et al., 2009; Lazennec and Richmond, 2010). Furthermore, CXCR4 and CCR5 act as co-receptors to mediate HIV entry into macrophages and T-cells (Berger et al., 1999). CCR5 (Xu et al., 2014) and CXCR4 (De Clercq, 2010) antagonists are on the market for the treatment of CCR5-tropic HIV infection and to promote mobilization of hematopoietic stem cells in transplant patients, respectively. Furthermore, the CCR4 monoclonal antibody Mogamulizumab has been approved in Japan for the treatment of adult T-cell leukemia-lymphoma (Yoshie and Matsushima, 2014).

### HERPESVIRUSES-ENCODED GPCRs

Human herpesviruses (HHVs) are double-stranded DNA viruses that establish a lifelong latent infection in the host (Vischer et al., 2006a,b). During latent infection, viral gene expression is highly suppressed and limited to a few genes that maintain the latent state and serve to evade immune detection. In the lytic phase, the majority of viral genes are expressed and viral DNA is replicated, leading to the production and release of infectious virions and the subsequent lysis of the host cell. Latent infections are usually asymptomatic. However, reactivation of the virus in immunocompromised patients (e.g., transplant recipients or AIDS patients) could lead to the development of serious pathologies (Cesarman, 2014b; Vischer et al., 2014). The HHVs are divided into α, β, and γ subfamilies based on their biological properties and sequence similarity (McGeoch et al., 2000). Members of the α subfamily are the human simplex virus (HSV)1 (HHV1), HSV2 (HHV2), and varicella zoster virus (HHV3). The subfamily of β herpesviruses consists of the human cytomegalovirus (HCMV; HHV5) and the Roseoloviruses (HHV6 and HHV7). Kaposi’s sarcoma-associated herpesvirus (KSHV; HHV8) and Epstein-Barr virus (EBV; HHV4) form the γ herpesvirus subfamily.

**FIGURE 5:**
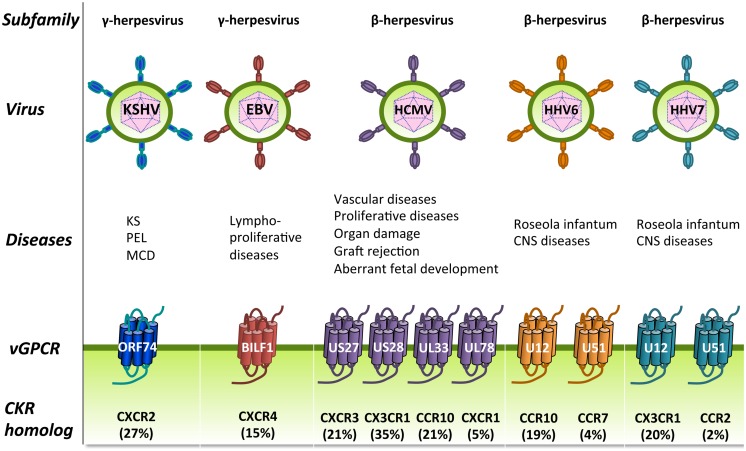
**Human herpesviruses (HHVs)-encoded GPCRs.** HHVs are divided into three subfamilies: the γ-herpesviruses (left), the β-herpesviruses (right), and the α-herpesviruses (not shown) and have been associated with several human diseases, including proliferative diseases. HHVs from the β and γ subfamilies encode one or more vGPCRs that show closest sequence identity to cellular chemokine receptors (percentage amino acid identity is shown between brackets). These vGPCRs have most likely been pirated from the human genome during viral evolution and function to modify cellular signaling. CKR, chemokine receptor; CNS, central nervous system; KS, Kaposi’s sarcoma; MCD, multicentric Castleman’s disease; PEL, Primary effusion lymphoma.

The β and γ herpesviruses encode homologs of human chemokine receptors and most of these receptors bind chemokines (**Figure 4**). These viral GPCRs (vGPCRs) have probably been derived from the host genome during evolution and modified to successfully redirect the functions of the host cells in favor of the virus. Some of these vGPCRs are involved in (proliferative) diseases (**Figure 5**; Vischer et al., 2006a,b, 2014; Slinger et al., 2011). The α herpesviruses do not encode GPCRs and are outside the scope of this review.

#### Kaposi’s sarcoma-associated herpesvirus

The KSHV genome is approximately 160 kbp long and encodes over 80 open-reading frames (Arvanitakis et al., 1996; Russo et al., 1996). KSHV is mainly spread via saliva, but could also be transmitted via contaminated blood and tissues transplants (Minhas and Wood, 2014) and infects endothelial cells via the interaction with integrins, heparin sulfates, and the ephrin receptor tyrosine kinase (RTK) A2 (Blackbourn et al., 2000; Akula et al., 2002; Kaleeba and Berger, 2006; Hahn et al., 2012; Garrigues et al., 2014). Moreover, B cells, monocytes/macrophages and dendritic cells are also permissive to KSHV infection (Blackbourn et al., 2000; Rappocciolo et al., 2006; Hassman et al., 2011; Dollery et al., 2014). The seroprevalence of KSHV depends on the geographical areas with infection rates up to 50% in Africa but only a few percent in the rest of the world (Hayward, 1999; Dedicoat and Newton, 2003). Latent infection of KSHV is often asymptotic. However, reactivation in immunosuppressed individuals can cause the development of proliferative disorders (**Figure 5**; Martin, 2007; Vischer et al., 2014). KSHV was first detected in 1994 in the Kaposi’s sarcoma (KS) lesions of an AIDS patient (Chang et al., 1994). KS is a highly vascularized neoplasm often found as red patches on the skin, but can also be presented in the oral cavity, lymph nodes and viscera. The tumor is composed of KSHV-infected spindle-shaped tumor cells of vascular and lymphatic endothelial origin, infiltrating inflammatory cells and red blood cells. Four different variants of KS are recognized. Classic KS affects middle-aged men of Mediterranean descent and is often benign. African endemic KS can be more aggressive and may also affect young children. AIDS-associated KS is the most aggressive form of KS and typically involves disseminated lesions that affect organs. Iatrogenic KS is associated with drugs-induced immunosuppression after transplantation (Radu and Pantanowitz, 2013). KSHV has also been associated with two rare lymphoproliferative disorders. Primary effusion lymphoma (PEL) is a HIV-associated non-Hodgkin’s lymphoma that arises in body cavities such as the pleural space, pericardium, and peritoneum. Dissemination of these lymphomas is not uncommon and the prognosis for patients with PEL is poor (Chen et al., 2007). Multicentric Castleman’s disease (MCD) involves the hyperproliferation of B cells in lymph nodes that may progress into lymphoma (Dittmer et al., 2012; Cesarman, 2014a).

KSHV encodes a single GPCR named ORF74, which shows highest sequence identity to human CXCR2 (**Figure 5**; Vischer et al., 2006b). Expression of ORF74 is detected in KS lesions (Cesarman et al., 1996; Staskus et al., 1997). Moreover, transgenic expression of ORF74 in mice is sufficient for the development of vascular KS-like lesions (Yang et al., 2000; Guo et al., 2003), indicating that ORF74 is a key player in the initiation of KS. ORF74 is a lytic gene which expression is regulated by the KSHV lytic master-switch protein ORF50 (Liang and Ganem, 2004). However, the role of a lytic gene in oncogenesis seems contradictory as cells expressing these genes eventually die when new virions are released. It has been proposed that immunosuppression or co-infection with HIV results in the dysregulated expression of ORF74 in non-lytic cells (Jham and Montaner, 2010). Furthermore, ORF74 is expressed in only a subset of KS tumor cells, but is able to transform neighboring cells by inducing the secretion of paracrine factors (Pati et al., 2001; Montaner et al., 2004; Martin and Gutkind, 2009). Indeed, selectively targeting ORF74-expressing cells in established tumors also resulted in apoptosis of adjacent cells that do not express ORF74 (Montaner et al., 2006).

#### Epstein-Barr virus

The genome of EBV has a size of 184 kbp and encodes approximately 84 open reading frames (Baer et al., 1984). EBV is primarily spread via saliva and widely distributed with 90% of the population being infected by their twenties (White et al., 2014). EBV infects B lymphocytes and epithelial cells via integrins and HLA class II molecules (Li et al., 1997; Haan et al., 2000; Dorner et al., 2010; Hutt-Fletcher and Chesnokova, 2010). EBV was one of the first discovered tumor viruses and was initially observed in cells derived from Burkitt’s lymphoma (Epstein et al., 1964). Later it was discovered that EBV is also involved in other cancer types such as Hodgkin’s lymphoma and nasopharyngeal carcinoma (**Figure 5**; Thompson and Kurzrock, 2004; Carbone et al., 2008).

EBV expresses a single GPCR named BILF1, which is expressed as an early lytic gene (Beisser et al., 2005). BILF1 shows highest sequence similarity to CXCR4 (**Figure 5**) and plays a role in escaping immune recognition by downregulating the surface expression of MHC class I proteins (Zuo et al., 2011; Griffin et al., 2013). MHC class I proteins present peptides derived from foreign proteins to cytotoxic T cells (Hewitt, 2003). As such, BILF1 reduces the activation of CD8+ T cells (Zuo et al., 2009). Furthermore, BILF1 inhibits the phosphorylated RNA-dependent protein kinase R (PKR), which plays a role in antiviral immune responses (Beisser et al., 2005).

#### Human cytomegalovirus

HCMV has the largest genome of the HHVs of approximately 230 kbp that is divided into a unique large (UL) and unique short (US) region. HCMV encodes over 200 open reading frames, but the exact number is depending on the strain (Murphy et al., 2003a,b; Stern-Ginossar et al., 2012). HCMV infects endothelial, epithelial, fibroblasts, and smooth muscle cells (Sinzger et al., 2008) via integrins (Feire et al., 2004, 2010) and growth factor receptors (Wang et al., 2003; Soroceanu et al., 2008) and is disseminated via latently infected monocytes. Differentiation of monocytes into macrophages leads to reactivation of HCMV and production of infectious virions (Streblow and Nelson, 2003). HCMV is found in the liver, gastrointestinal track, lungs, retina, and brain and widely spread among the population with a seroprevalence ranging from 50–100% (Gandhi and Khanna, 2004; Bate et al., 2010; Cannon et al., 2010). Primary infection or reactivation of HCMV in immunocompromised hosts can cause severe and fetal conditions such as damage to HCMV-positive organs (the liver, lungs, brain, and retina; **Figure 5**; Landolfo et al., 2003). Furthermore, HCMV is associated with vascular diseases such as atherosclerosis, inflammatory, and autoimmune diseases (Gombos et al., 2013). HCMV infection during pregnancy might cause severe problems to the unborn child, such as neurosensory hearing loss or mental retardation (Carlson et al., 2010). Primary HCMV infection in transplantation patients may cause graft rejection and diseases to the donor organ (Cainelli and Vento, 2002; Ishibashi et al., 2011). Finally, HCMV infection is associated with various malignancies including colon cancer (Mariguela et al., 2008; Bongers et al., 2010) and glioblastoma (Cobbs, 2013). HCMV has been proposed to act as an oncomodulator rather than an oncogenic virus. HCMV preferentially infects cancer cells and regulates the expression of oncogenic and tumor suppressor genes (Michaelis et al., 2009; Soderberg-Naucler and Johnsen, 2012).

HCMV encodes four GPCRs: US27, US28, UL33, and UL78. These vGPCRs display highest sequence identity to human chemokine receptors CXCR3, CX3CR1, CCR10, and CXCR1, respectively (**Figure 5**; Vischer et al., 2006b). US28 (Zipeto et al., 1999) and UL78 (Michel et al., 2005) are expressed early after HCMV infection whereas US27 (Margulies and Gibson, 2007) and UL33 (Bodaghi et al., 1998) are expressed with late kinetics. In addition, the protein products of the HCMV-encoded GPCRs are found in viral particles (Bodaghi et al., 1998), which indicates that these vGPCRs might contribute to viral dissemination. UL78 (O’Connor and Shenk, 2012) and US27 (O’Connor and Shenk, 2011) have been proposed to play a role in the viral life cycle and dissemination. A specific role for US28 in the oncomodulatory properties of HCMV has been postulated due to the proliferative, pro-angiogenic, and pro-inflammatory signaling of US28 (Maussang et al., 2006). Moreover, US28 was detected in glioblastoma specimens from patients (Slinger et al., 2010; Soroceanu et al., 2011). Furthermore, US28 induces the migration of inflammation-associated cells that are often involved in vascular diseases, such as vascular smooth muscle cells and macrophages (Vomaske et al., 2009). In addition, US28 acts as a co-receptor for HIV entry (Pleskoff et al., 1997).

#### Roseoloviruses

Roseoloviruses consist of three highly related species: HHV6A, HHV6B, and HHV7. These species have a similar genomic organization, but differ in their epidemiologic and biological characteristics. The sequence identity between HHV6A and 6B is 90% and the genome size of both variants is approximately 160 kbp (Dominguez et al., 1999). HHV6A and 6B encode 110 and 119 open reading frames, respectively (Caselli and Di Luca, 2007). The genome of HHV7 has a size of approximately 150 kbp and encodes 84 open reading frames (Caselli and Di Luca, 2007). HHV6 is probably transmitted via saliva (Tang and Mori, 2010) and enters cells via the interaction with CD46 (Tang and Mori, 2010). HHV6 establishes latency mainly in monocytes, but also in bone marrow progenitors, the salivary glands, and the central nervous system, but replicates most efficiently in CD4+ T cells (De Bolle et al., 2005). HHV7 persists latently in T lymphocytes and uses CD4 for cell entry (Lusso et al., 1994). Infection with Roseoloviruses often occurs during early childhood and seroprevalence in adults is almost 100% (Emery and Clark, 2007). Primary infection with HHV6 (and less common with HHV7) in children can lead to Roseola infantum, an illness characterized by fever and rash (**Figure 5**; Tanaka et al., 1994; Tang et al., 2010). Furthermore, reactivation of Roseoloviruses in immunocompromised hosts is associated with diseases of the central nervous system such as encephalitis, encephalopathy, and multiple sclerosis, but also with pneumonitis, hepatitis, bone marrow suppression, and rejection of transplanted organs and even death (Campadelli-Fiume et al., 1999; Schwartz et al., 2014).

Both HHV6 and HHV7 encode two GPCRs: U12 and U51. HHV6-U12 shares highest sequence similarity with CCR10, HHV7-U12 with CX3CR1, HHV6-U51 with CCR7 and HHV7-U51 with CCR2 (**Figure 5**; Vischer et al., 2006b). U51 is expressed early after viral infection (Menotti et al., 1999), whereas U12 is a late gene expressed during lytic infection (Isegawa et al., 1998).

## vGPCRs MODULATE CELLULAR SIGNALING

To persist in the host and to replicate and spread themselves is of vital importance for viruses. To achieve this, viruses such as the herpesviruses developed multiple strategies. For example, a large amount of viral gene products of herpesviruses is dedicated to evade antiviral immune responses (Griffin et al., 2010). Most of the vGPCRs show highest sequence identity to human chemokine receptors. Considering the functions of chemokine receptors, herpesviruses might use vGPCRs for immune evasion and/or viral replication and dissemination by inducing proliferation and chemotaxis of infected cells. vGPCRs have developed multiple ways to modulate cellular signaling for the benefit of the virus. Hijacking of human chemokines and G proteins by vGPCRs have been the subject of many studies. However, exploiting human cellular trafficking proteins or modulating the function of human receptors from the GPCR or receptor tyrosine kinase (RTK) class might be alternative strategies of the virus to modulate cellular responses in favor of the virus.

### vGPCRs HIJACK CHEMOKINES

In contrast to their human counterparts, most vGPCRs are constitutively active (see also “vGPCRs Hijack Human G Proteins” and “Molecular Determinants of the Constitutive Activity of Viral GPCRs”), meaning that they can activate signaling pathways in a ligand-independent manner. However, most vGPCRs are also able to bind human and viral chemokines that modulate this constitutive activity in some cases or to activate ligand-dependent signaling. In contrast to human chemokine receptors, vGPCRs can bind chemokines from several families (**Figure 4**).

#### Chemokine binding to KSHV-encoded ORF74

ORF74 binds a broad range of CXC chemokines that also bind to its closest human homolog CXCR2: CXCL1-3 and CXCL5-8 (Rosenkilde et al., 1999). ORF74 binds also to CXCL4, CXCL10 (CXCR3 agonist), and CXCL12 (CXCR4 agonist), which do not bind to CXCR2 (Gershengorn et al., 1998; Rosenkilde et al., 1999). Furthermore, ORF74 binds to CCL1 and CCL5 (**Figure 4**; Arvanitakis et al., 1997). These human chemokines modulate the constitutive activity of ORF74 toward different signaling pathways that are described in Section “G Protein-Dependent ORF74 Signaling”. CXCL1 and CXCL3 are full agonists, whereas CXCL2 acts as a partial agonist. CXCL4, CXCL5, CXCL7, and CXCL8 behave as low-potency agonists. CXCL10 and CXCL12 are full inverse agonists and CXCL6 is a partial inverse agonist (Geras-Raaka et al., 1998b,c; Gershengorn et al., 1998; Rosenkilde et al., 1999). Furthermore, KSHV encodes three viral chemokines: vCCL1, vCCL2, and vCCL3 (**Figure 4**). vCCL2 acts as a partial inverse agonist for ORF74 (Geras-Raaka et al., 1998b), as an antagonist for several human chemokine receptors expressed on T helper 1 (T_H_1) cells and as an agonist for CCR3 and CCR8 expressed on T_H_2 cells (Nicholas, 2010). vCCL1 and vCCL3 are agonists of human CCR8 and CCR4, respectively, and thereby attract T_H_2 cells, which are less effective against pathogens as compared to T_H_1 cells (Coscoy, 2007).

Similar to human chemokine receptors (Scholten et al., 2012), the N-terminus of ORF74 is essential for high affinity chemokine binding (Ho et al., 1999). The N-terminus contains two tyrosine (Y) residues, which are post-translationally modified by sulfate groups. Aspartic acid-substitution of these Y residues diminished sulfation of ORF74 but did not affect constitutive signaling of ORF74. However, CXCL1 binding to this mutant was impaired, whereas CXCL10 binding was preserved compared to wild type (WT)-ORF74 (Feng et al., 2010). This suggests that CXCL1 and CXCL10 differentially interact with the N-terminus of ORF74. Interestingly, the ORF74 mutant lacking sulfo-tyrosines did not form tumors in a xenograft mouse model, as compared to WT-ORF74, suggesting that CXCL1-induced signaling of ORF74 is essential for tumor formation in mice (Feng et al., 2010). Similar conclusions were drawn from the transgenic expression of an N-terminal deletion mutant, that is constitutively active but unable to bind chemokines, and did not develop KS-like lesions in mice (Holst et al., 2001). Furthermore, the double mutant ORF74-R^5.35(208)^H/R^5.39(212)^H is constitutively active and responsive to inverse agonists, but not to agonists. When this mutant is expressed in transgenic mice, a smaller fraction of mice develop KS-like disease and with a reduced severity compared to mice expressing WT-ORF74 (Holst et al., 2001).

#### Chemokine binding to EBV-encoded BILF1

No ligands have hitherto been identified for BILF1 (**Figure 4**). Therefore, BILF1 has been classified as an orphan receptor.

#### Chemokine binding to HCMV-encoded GPCRs

US28 binds CX3CL1, several chemokines from the CC family (e.g., CCL2 and CCL5; Gao and Murphy, 1994; Kuhn et al., 1995; Kledal et al., 1998) and KSHV-encoded vCCL2 (**Figure 4**; Kledal et al., 1997). Human CCL5 and CX3CL1 differentially interact with the US28 N-terminus (Casarosa et al., 2005) and differentially modulate (constitutive) US28 signaling, as further discussed in Section “G Protein-Dependent Signaling of HCMV-Encoded GPCRs” (Vomaske et al., 2009).

The medium of HCMV-infected fibroblasts contains lower levels of CCL2 and CCL5 as compared to the medium of uninfected fibroblasts, which is not due to decreased transcriptional activity (Michelson et al., 1997; Bodaghi et al., 1998) or degradation by soluble proteases (Michelson et al., 1997), but is instead the result of the co-internalization of chemokines with US28 (Michelson et al., 1997; Bodaghi et al., 1998; Billstrom et al., 1999). This chemokine scavenging might be an immune evasion strategy to regulate chemokine levels available for human chemokine receptors and subsequently limit attraction of surrounding immune cells at sites of infection. Indeed, the supernatant from HCMV-infected fibroblasts is unable to induce migration of monocytes (Randolph-Habecker et al., 2002). On the other hand, monocyte adhesion to a monolayer of endothelial cells that express US28 through retroviral transduction is not inhibited as compared to cells transduced with empty virus, indicating that chemokine scavenging by US28 is insufficient to affect monocyte adhesion (Boomker et al., 2006).

Furthermore, binding of US28 to membrane-tethered CX3CL1 of the host cell promotes cell-to-cell contact and might consequently facilitate viral dissemination (Kledal et al., 1998). Indeed, an US28-deletion mutant of HCMV (HCMV-ΔUS28) shows a significant decrease in cell-to-cell infection of epithelial cells, as compared to WT-HCMV (Noriega et al., 2014).

The other three HCMV-encoded GPCRs US27, UL33, and UL78 are classified as orphan receptors as they do not seem to interact with chemokines or other ligand types as of yet.

Besides viral GPCRs, HCMV also encodes two viral chemokines. vCXCL1 induces Ca^2+^ mobilization in L1.2 cells stably transfected with CXCR1 or CXCR2 (**Figure 4**), whereas no Ca^2+^ response was observed in cells expressing any of the other human chemokine receptors tested (Luttichau, 2010). No receptor for vCXCL2 has hitherto been identified and it remains to be investigated whether these HCMV-encoded chemokines are able to bind viral GPCRs.

#### Chemokine binding to Roseoloviruses-encoded GPCRs

U12 and U51 encoded by HHV6 and HHV7 bind to different chemokines. HHV6-U12 binds to CCL2-5, while HHV7-U12 binds to CCL17, CCL19, CCL21, and CCL22. Likewise, HHV6-U51 binds to several CC-chemokines, CX3CL1, XCL1 (Catusse et al., 2008), and KSHV-encoded vCCL2 (Milne et al., 2000), whereas HHV7-U51 only binds CC-chemokines (**Figure 4**). Signaling mediated by HHV6-U12 (Isegawa et al., 1998) and HHV7-U12 (Tadagaki et al., 2005) is dependent on chemokines and the constitutive activity of HHV6-U51 is differentially regulated by chemokines, as further discussed in Section “G Protein-Dependent Signaling of Roseoloviruses-Encoded GPCRs”. Besides chemokine binding, U51 may subvert recognition by the immune system by decreasing CCL5 concentrations at the transcriptional level in stably transfected epithelial and erythroleukemia cells and in HHV6-infected T lymphocytes (Milne et al., 2000; Catusse et al., 2008).

HHV6 encodes the chemokine vCCL4, which binds to human CCR2 and activates Ca^2+^ mobilization and migration of CCR2-expressing L1.2 cells (Luttichau et al., 2003). Hence, HHV6 might utilize vCCL4 to attract CCR2-expressing cells (i.e., monocytes/macrophages) for infection and to establish latency (Luttichau et al., 2003).

### vGPCRs HIJACK HUMAN G PROTEINS

Most agonist-occupied GPCRs activate downstream signaling via the coupling and activation of heterotrimeric G proteins. The crystal structure of the active β_2_ adrenergic receptor (β_2_AR) in complex with Gα_s_ revealed that R^3.50(131^; Ballesteros–Weinstein residue numbering (Ballesteros and Weinstein, 1995) followed by UniProt residue numbering) of β_2_AR packs against Gα_s_ (Rasmussen et al., 2011), suggesting a direct interaction. R^3.50^ is part of the DRY (aspartic acid–arginine–tyrosine) motif, which is located at the boundary of TM3 and ICL2 and plays a key role in G protein activation. The DRY motif is one of the most conserved motifs among rhodopsin-like GPCRs with R^3.50^ being the most conserved (96%; Mirzadegan et al., 2003). Mutation of R^3.50^ impairs G protein signaling of many GPCRs (Rovati et al., 2007).

#### G protein-dependent ORF74 signaling

In contrast to its human homolog CXCR2, ORF74 couples constitutively and promiscuously to Gα_i_, Gα_12/13_, and presumably Gα_q_ [the latter was suggested from the PTX-insensitive activation of PLC in transiently transfected COS-7 cells (Shepard et al., 2001; Smit et al., 2002; Cannon and Cesarman, 2004; Rosenkilde et al., 2004; Verzijl et al., 2004)]. G protein coupling leads to the constitutive activation of a variety of signal transduction cascades that contribute to the oncogenic properties of ORF74. For example, ORF74 constitutively activates MAP kinases such as ERK1/2, p38, and JNK in both a PTX-sensitive and -insensitive manner (Bais et al., 1998; Munshi et al., 1999; Sodhi et al., 2000; Smit et al., 2002), which may promote the expression of growth-promoting genes. Interestingly, ERK is activated in ORF74-expressing COS-7 cells (Smit et al., 2002) but not in HEK293T cells (Bais et al., 1998), showing cell-type-dependency of ORF74 signaling. Furthermore, the activation of PI3K and Akt protects cells from apoptosis (Montaner et al., 2001) and may therefore contribute to the survival of KSHV-infected cells. Constitutive Akt activation by ORF74 leads to activation of the TSC2/mTOR pathway in transiently transfected COS-7 cells (Sodhi et al., 2006). Inhibition of mTOR activity leads to tumor regression in a KS mouse model, whereas mTOR overexpression was sufficient to render endothelial cell oncogenic when injected in mice (Sodhi et al., 2006). Additionally, ORF74 constitutively activates members of the Rho family of small guanosine triphosphatases (GTPases) such as RhoA (Shepard et al., 2001; Martin et al., 2007) and Rac1 (Montaner et al., 2004) in transfected HEK293T, NIH-3T3, and porcine aortic endothelial cells via Gα_12/13_ proteins. Rac1 is overexpressed in spindle cells from KS biopsies and expression of a constitutively active Rac1 (Ma et al., 2009) or RhoA (Martin et al., 2007) in transgenic mice causes the development of KS-like tumors, whereas inhibition of Rac1 (Montaner et al., 2004) or knockdown of RhoA expression (Martin et al., 2007) reduces ORF74 tumorogenesis *in vivo*. Most of these constitutively activated signaling pathways (i.e., PLC, ERK, Akt, and NFAT activation) are modulated by chemokines as described in Section “Chemokine Binding to KSHV-Encoded ORF74” (Rosenkilde et al., 1999; Smit et al., 2002).

These pathways lead to the constitutive activation of numerous transcription factors including nuclear factor κ-light-chain-enhancer of activated B cells (NF-κB), NFAT, CRE, activator protein 1 (AP1) and hypoxia-inducible factor 1α (HIF-1α) in different cell lines [e.g., COS-7, HEK293, T cells, and monocytes, but also more relevant cells such as KSHV-positive primary B cells derived from KS patients (Azzi et al., 2013), the KS-derived endothelial cell line KSIMM, primary endothelial cells (Pati et al., 2001), and PEL cells (Cannon and Cesarman, 2004)]. Constitutive NF-κB activation is partly inhibited by PTX, showing a contribution of Gα_i/o_ coupling (Cannon and Cesarman, 2004; Verzijl et al., 2004). However, the agonistic effects of the murine chemokines CXCL1 and CXCL2 were insensitive to PTX, suggesting that agonists induce a switch in G protein coupling (Verzijl et al., 2004). HIF-1α regulates the expression of vascular endothelial growth factor (VEGF), which contributes to the angiogenic potential of ORF74 (Sodhi et al., 2000). AP-1, NFAT, and NF-κB are important mediators of the expression of cytokines such as CXCL1, CXCL8, and IL-6 in HEK293T cells, monocytes (Schwarz and Murphy, 2001), endothelial cells and KSIMM cells (Pati et al., 2001), which produce an inflammatory environment that promotes transformation of cells and contributes to KS (Cesarman et al., 2000). These secreted factors promote proliferative and pro-angiogenic signaling in an autocrine, but also paracrine manner by activating neighboring cells that do not express ORF74 (Sodhi et al., 2004a). In addition, these secreted paracrine factors might attract host cells that are potentially infected by new KSHV viruses and thereby contribute to viral dissemination.

The DRY motif is less conserved among vGPCRs and most variety is observed within D^3.49^ (Jensen et al., 2012). Indeed, ORF74 contains a VRY motif and mutation of the DRY motif of CXCR2 into VRY introduces constitutive activity to this human homolog of ORF74 constitutively active (Burger et al., 1999). Reciprocally, introducing a DRY motif in ORF74 did not have major effects on its signaling properties (Rosenkilde et al., 2000). On the other hand, substitution of R^3.50(143)^ with alanine results in a non-functional mutant of ORF74 (Ho et al., 2001) that lacks oncogenic potential (Sodhi et al., 2004b; Chaisuparat et al., 2008). Hence, G protein-dependent signaling is essential for ORF74 Kaposi’s sarcomagenesis. Interestingly, the equine herpesvirus 2 (EHV2)-encoded ORF74 lacks the conserved R^3.50^ but is functionally and constitutively coupled to Gα_i_ in HEK293T cells (Rosenkilde et al., 2005).

#### G protein-dependent BILF1 signaling

BILF1 constitutively activates the transcription factor NF-κB and inhibits CRE via G_i_ proteins in transfected COS-7 cells (Beisser et al., 2005; Paulsen et al., 2005). In contrast, BILF1 is unable to constitutively modulate NF-κB-mediated gene activation but activates CRE-mediated transcription in Burkitt’s lymphoma and lymphoblastoid B cells (Beisser et al., 2005), showing that BILF1 signaling can be cell type dependent. BILF1 exhibits the sequence EKT instead of the DRY motif. BILF1-k^3.50(122)^A is unable to inhibit the forskolin-induced increase in cAMP (Lyngaa et al., 2010), but still induces tumor growth in a xenograft mouse model (Lyngaa et al., 2010). This suggests that G protein-independent signaling contributes to tumor development. Introduction of a DRY motif yielded a less active mutant of BILF1 compared to WT-BILF1 with respect to cAMP signaling (Lyngaa et al., 2010).

#### G protein-dependent signaling of HCMV-encoded GPCRs

US28 constitutively activates proliferative, pro-survival, and pro-inflammatory signaling pathways. For example, US28 constitutively activates PLC in transfected COS-7 or NIH-3T3 cells (Casarosa et al., 2001; Waldhoer et al., 2002; Maussang et al., 2006) and HCMV-infected smooth muscle cells and U373 glioblastoma cells (Miller et al., 2012). Constitutive signaling via Gα_q_ and Gα_i_ proteins eventually leads to the activation of transcription factors such as NFAT, NF-κB, CRE, and SRF (McLean et al., 2004), resulting in the upregulation of cyclinD1 (Maussang et al., 2006), VEGF (Maussang et al., 2006), COX-2 (Maussang et al., 2009a), β-catenin (Langemeijer et al., 2012), and IL-6 (Slinger et al., 2010) in COS-7, HEK293T, NIH-3T3 cells, and HCMV-infected glioblastoma cells. Secreted IL-6 activates the proliferative IL-6/JAK1/STAT3 signaling axis (Slinger et al., 2010).

US28 also signals in a ligand-dependent manner. CCL5 is required for the US28-mediated activation of RhoA (Melnychuk et al., 2004), focal adhesion kinase (FAK), and ERK (Vomaske et al., 2009) via Gα_12/13_ proteins in smooth muscle cells, mouse fibroblasts and U373 glioblastoma cells infected with adenoviruses expressing US28, whereas CX3CL1 activates FAK and ERK via Gα_q_ in fibroblasts. US28 adenovirus-expressing smooth muscle cells migrate toward CCL5, whereas CX3CL1 antagonizes this effect (Vomaske et al., 2009). On the other hand, CX3CL1 (but not CCL5) induces migration of US28-expressing macrophages (Vomaske et al., 2009), showing that the effect of chemokines can be cell type dependent. The migration of HCMV-infected cells may have important implications for viral spread but also in cardiovascular diseases such as atherosclerosis where macrophages and smooth muscle cells migrate into the atherosclerotic plaques. Furthermore, both CCL5 and CX3CL1 promote US28-dependent Ca^2+^ mobilization in a PTX-insensitive manner in HCMV-infected smooth muscle cells, but not in U373 glioblastoma cells (Miller et al., 2012). Moreover, CCL5 further enhances the US28-mediated invasiveness of glioma cells and primary glioblastoma cultures (Soroceanu et al., 2011), showing the relevance of ligand-induced signaling in US28-associated pathologies.

In contrast to the agonistic effects of CX3CL1 on FAK, ERK, and Ca^2+^ signaling, this chemokine can also act as an inverse agonist as it decreases constitutive PLC and NFAT activation in transiently transfected COS-7 and HEK293 cells (Casarosa et al., 2001; McLean et al., 2004). However, CX3CL1 increases PLC activation in the absence of the C-terminus of US28 (Waldhoer et al., 2003). It has been argued that the endocytosis of US28 camouflages the agonistic properties of CX3CL1 and blocking endocytosis by removing the C-terminus unmasks CX3CL1 agonism (Waldhoer et al., 2003).

Since US28 has been linked to proliferative and cardiovascular diseases, inverse agonists targeting the constitutive activity and chemokine binding to US28 might be attractive therapeutic agents. The first identified small molecule inhibitor of US28, VUF2274, inhibits constitutive PLC activation, and CCL5 binding to US28 in transfected and HCMV-infected cells (Casarosa et al., 2003b). Furthermore, VUF2274 also inhibits HIV entry in US28-expressing cells. Later, analogs of VUF2274 and other scaffolds were identified to inhibit CCL5 binding and constitutive signaling of US28 (Hulshof et al., 2005, 2006; Vischer et al., 2010; Kralj et al., 2013, 2014). Similar to CX3CL1, VUF2274 acts as agonists on the C-terminal truncated mutant US28-Δ300. Interestingly, other small molecules retained their inhibitory properties on US28-Δ300 (Tschammer, 2014).

The DRY mutant US28-R^3.50(129)^A is unable to activate PLC and several transcription factors (Maussang et al., 2006, 2009a; Stropes and Miller, 2008; Slinger et al., 2010). However, US28-R^3.50(129)^A-expressing cells are still able to form tumors in nude mice, albeit at later time points as compared to WT-US28-expressing cells (Maussang et al., 2006). This indicates that G protein-independent signaling also contributes to the oncomodulatory properties of US28.

UL33 also possesses a conserved DRY motif and promiscuously couples to Gα_i_, Gα_q_, and Gα_s_ proteins to constitutively activate PLC, p38, and CREB in COS-7 cells (Casarosa et al., 2003a). Although US27 and UL78 are required for viral dissemination (O’Connor and Shenk, 2011) and viral entry (O’Connor and Shenk, 2012), these receptors have long been considered ‘silent’ as no signaling was detected. However, it was recently shown that US27 promotes cell proliferation, cell survival and the expression of a limited number of genes (e.g., the pro-survival factor Bcl-x and AP-1) when expressed in HEK293T, HeLa, and COS-7 cells (Lares et al., 2013; Tu and Spencer, 2014). The DRY mutant US27-R^3.50(128)^A decreased cell proliferation comparable to rates observed in mock-transfected cells (Tu and Spencer, 2014). However, it remains to be investigated if US27 signals via G proteins.

#### G protein-dependent signaling of Roseoloviruses-encoded GPCRs

HHV6-U12 and HHV7-U12 increase intracellular Ca^2+^ concentrations via a PTX-insensitive pathway in a ligand-dependent manner in transfected erythroleukemia cells (Isegawa et al., 1998; Nakano et al., 2003; Tadagaki et al., 2005). Furthermore, CCL19 and CCL21 (but not CCL17 and CCL22) induce HHV7-U12-mediated chemotaxis of Jurkat cells (Tadagaki et al., 2005). HHV6-U51 constitutively activates PLC and inhibits CRE in COS-7 cells via Gα_q_, as was shown by the inhibiting effect of the co-expressed Gα_q/11_ scavenger GRK2 (Fitzsimons et al., 2006; Catusse et al., 2008; see “Desensitization and Intracellular Receptor Trafficking of Viral GPCRs”). Interestingly, CCL2, CCL5, and CCL11 counteract constitutive HHV6-U51-induced inhibition of CRE activity in a PTX-sensitive manner, whereas only CCL5 increases constitutive PLC activation and promotes Ca^2+^ mobilization in a PTX-insensitive manner. These differential effects of the chemokines might be explained by coupling of HHV6-U51 to distinct G protein subtypes (Fitzsimons et al., 2006). The chemokines that bind to HHV7-U51 induce Ca^2+^ mobilization most likely also via Gα_q_, but do not promote chemotaxis of Jurkat cells (Tadagaki et al., 2005).

### MOLECULAR DETERMINANTS OF THE CONSTITUTIVE ACTIVITY OF VIRAL GPCRs

According to the ternary complex model (De Lean et al., 1980), an agonist is required to activate a GPCR. However, the discovery of constitutively active GPCRs that show signaling in the absence of agonists led to the extended ternary complex model (Samama et al., 1993). In this revised model, a GPCR exists in equilibrium between inactive and active conformations. Agonists shift the equilibrium toward active receptors, whereas for constitutively active GPCRs already a larger receptor fraction is in the active conformation. Inverse agonists stabilize the inactive conformation and consequently inhibit constitutive activity. This model was later modified to the cubic ternary complex model to incorporate the notion that G proteins can also bind to inactive receptors (Weiss et al., 1996). More than 60 WT GPCRs are reported to show constitutive activity that is inhibited by inverse agonists (Seifert and Wenzel-Seifert, 2002). However, most of these receptors are studied in recombinant cell lines using expression levels that exceed physiological levels and the extent of constitutive activity varies with cellular background (Seifert and Wenzel-Seifert, 2002). On the other hand, constitutive activity of some GPCRs has also been observed in native cells or tissues (Seifert and Wenzel-Seifert, 2002). In addition, naturally occurring mutations in some GPCRs increase constitutive activity and are associated with human diseases (Smit et al., 2007; Tao, 2008).

#### Constitutive activity of ORF74

The constitutive activity of ORF74 is probably attributed to mutations in residues that are highly conserved in other GPCRs and that may stabilize the inactive conformation. For example, the interaction between R^3.50^, D/E^3.49^, and D/E^6.30^ is known as the ionic lock (Ballesteros et al., 2001). Charge-neutralizing mutations in D/E^3.49^ or D/E^6.30^ increase the constitutive activity of many human GPCRs (Scheer et al., 1996; Kim et al., 1997; Ballesteros et al., 2001; Montanelli et al., 2004). Although the exact molecular basis for the constitutive activity of ORF74 is not clear, D/E^3.49^, and D/E^6.30^ are substituted in ORF74 by V^3.49(142)^ and R^6.30(246)^, respectively, which might possibly results in the disruption of the ionic lock. Although the V^3.49(142)^D mutation did not decrease the constitutive activity of ORF74 (Rosenkilde et al., 2000; Ho et al., 2001), restoring the ionic lock by introducing an aspartic acid or glutamic acid at R^6.30(246)^ in ORF74 has to our best knowledge not been investigated.

The highly conserved W^6.48^ in TM6 of GPCRs undergoes a conformational change from pointing toward TM7 to pointing toward TM5 upon receptor activation. This results in the movement of the end of TM6 away from TM3 and consequently disrupts the ionic lock. This is known as the transmission switch and is likely a common activation mechanism for most GPCRs (Trzaskowski et al., 2012). In ORF74, W^6.48^ is mutated to C^6.48(264)^, which might influence the transmission switch. Another example is N^7.49^ from the highly conserved NPxxY motif in GPCRs that forms a network of hydrogen bonds with D^2.50^ (Urizar et al., 2005). In ORF74 these residues are mutated to V^7.49(310)^ and S^2.50(93)^, respectively, possibly resulting in the disruption of the water-mediated hydrogen bonding network and stabilizing the active conformation of TM7. Interestingly, the constitutive activity of the S^2.50(93)^D and the V^7.49(310)^N mutants of ORF74 are unaltered compared to WT-ORF74 (Rosenkilde et al., 2000). However, mutation of both residues in the S^2.50(93)^D/V^7.49(310)^N double mutant might be required to stabilize the inactive conformation of ORF74. Moreover, an H-bonding network between helix8 and residues of TM2 and TM7 of ORF74 was proposed to stabilize the end of TM7 (Verzijl et al., 2006). Disruption of helix8 by deletion or point mutations [R^7.61(322)^W and Q^7.62(323)^P] distorts this network and results in inactive mutants (Verzijl et al., 2006). Finally, the L^2.48(91)^D and L^2.51(94)^D (but not N^2.49(92)^D and S^2.50(93)^D) mutants of ORF74 are deficient in constitutive activity but still signal in response to chemokines. In contrast to N^2.49(92)^ and S^2.50(93)^, L^2.48(91)^ and L^2.51(94)^ are predicted to face the lipid bilayer. Possibly, the substitution of hydrophobic residues with charged residues facing the cell membrane destabilizes the active conformation of ORF74 (Rosenkilde et al., 2000). Transgenic mice carrying the L^2.48(91)^D mutant of ORF74 fail to develop KS-like lesions (Holst et al., 2001). This shows that the constitutive activity of ORF74 plays a key role in KS.

#### Constitutive activity of other vGPCRs

BILF1 (Paulsen et al., 2005), US28 (Casarosa et al., 2001), UL33 (Waldhoer et al., 2002), and HHV6-U51 (Fitzsimons et al., 2006) are all constitutively active. As for ORF74, the molecular basis for this constitutive activity is not well understood. For example, the residues involved in the ionic lock, the transmission switch and the NPxxY motif are all conserved in US28. It has been proposed that A^3.35(114)^ might underlie the constitutive activity of US28 as it would be too small to interact with residues from TM2, TM3, and TM7 to stabilize the inactive conformation (Montaner et al., 2013). However, no mutational studies on US28-A^3.35(114)^ have been performed to verify these predictions.

### DESENSITIZATION AND INTRACELLULAR RECEPTOR TRAFFICKING OF VIRAL GPCRs

**FIGURE 6:**
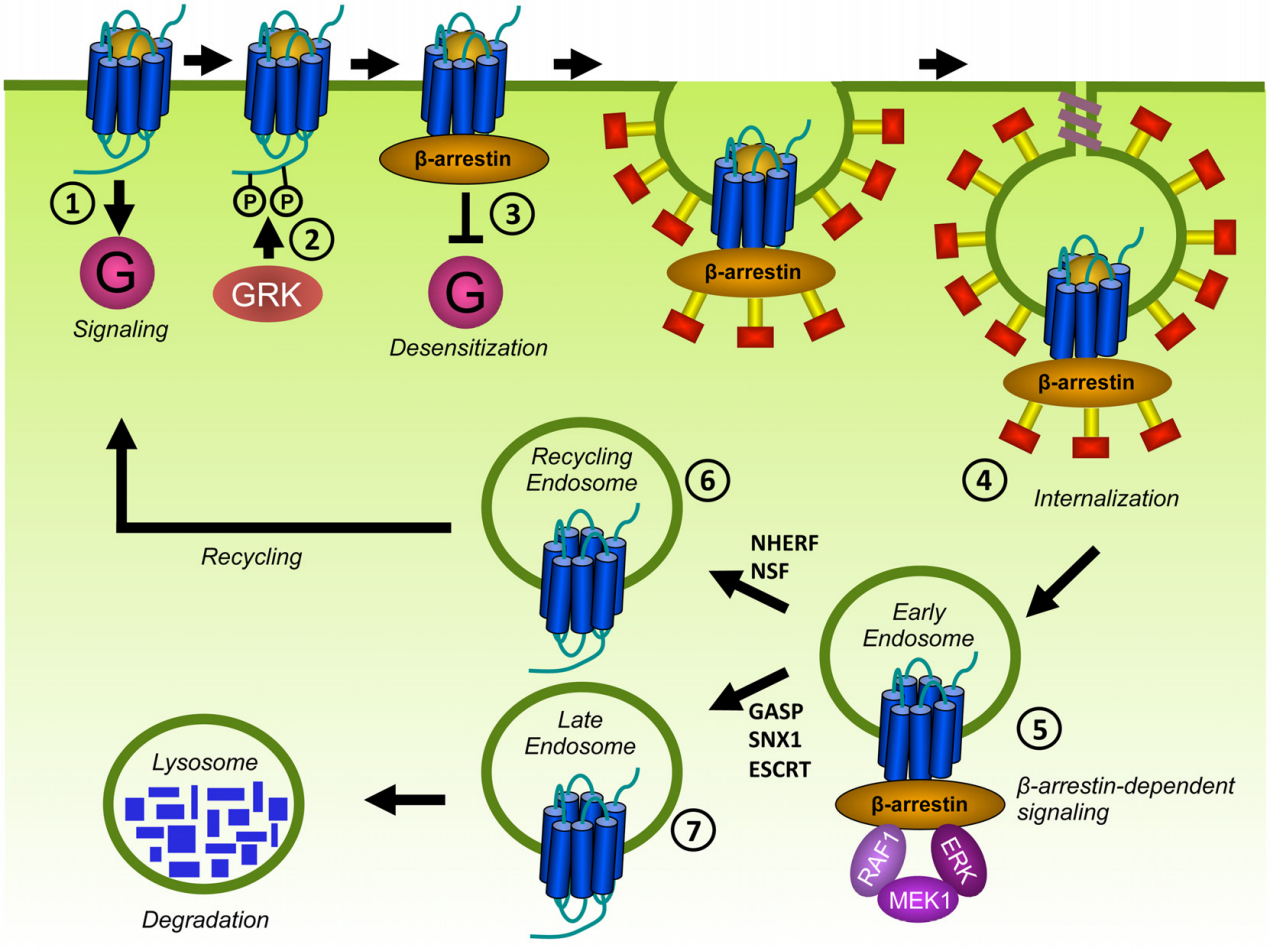
**Desensitization and trafficking of GPCRs.** Upon ligand binding, GPCRs traditionally signal via G proteins **(1)**. In addition, GPCRs are phosphorylated on S and T residues in their C-terminus or ICLs by GRKs **(2)**. β-arrestins bind to phosphorylated GPCRs and prevent further coupling of G proteins, a process known as desensitization **(3)**. β-arrestins target phosphorylated GPCRs for endocytosis via clathrin-coated pits (CCPs) by scaffolding proteins of the internalization-machinery **(4)**. Internalized receptors may activate β-arrestin-dependent signaling **(5)**. Internalized GPCRs are subsequently sorted to recycling endosomes **(6)** or to lysosomes for degradation **(7)**.

After activation, the temporal and spatial signaling of GPCRs is controlled by desensitization and internalization (**Figure 6**). GPCR desensitization involves the phosphorylation of serine (S) and threonine (T) residues in the C-terminus or sometimes the ICLs of GPCRs (Nakamura et al., 1998; Kim et al., 2001; Liang et al., 2002; Trester-Zedlitz et al., 2005; Watari et al., 2014) by G protein-coupled receptor kinases (GRKs) and the subsequent inhibition of G protein activation. GRKs are activated upon docking to active GPCRs and thus regulate homologous desensitization of GPCRs (Gurevich et al., 2012). The GRK family is composed of 7 members (GRK1-7). Whereas GRK1, GRK7 (retina; Hisatomi et al., 1998) and GRK4 (testis; Premont et al., 1996) display tissue-specific expression, GRK2, GRK3, GRK5, and GRK6 are ubiquitously expressed throughout the body and phosphorylate the majority of GPCRs. GRK2, and GRK3 contain a N-terminal regulator of G protein signaling homology (RH) domain which enables them to selectively interact with activated Gα_q_ (Ferguson, 2007) and allows GRK2 and GRK3 to inhibit Gα_q_-mediated signaling of several GPCRs independently of receptor phosphorylation (Carman et al., 1999; Sallese et al., 2000; Dhami et al., 2004; Iwata et al., 2005; Giannotta et al., 2012). Second messenger-dependent protein kinases such as PKA and PKC are able to phosphorylate also inactive GPCRs and contribute to heterologous desensitization (Kelly et al., 2008). Other S/T kinases involved in GPCR phosphorylation include Akt (Lee et al., 2001; Doronin et al., 2002), casein kinase 1 (CK1; Tobin et al., 1997; Luo et al., 2008) and CK2 (Hanyaloglu et al., 2001).

Phosphorylated GPCRs recruit β-arrestins to the plasma membrane (**Figure 6**). The arrestin family is composed of four members. Although arrestin1 and arrestin4 are specifically expressed in the visual system to regulate rhodopsin, β-arrestin1 (also known as arrestin2), and β-arrestin2 (arrestin3) bind to the majority of non-visual GPCRs. β-arrestins sterically hinder the coupling to G proteins and subsequently inhibit further activation of G protein-mediated signaling. Upon binding to phosphorylated GPCRs, β-arrestins undergo conformational changes. This results in the exposure of domains that interact with components of the endocytic machinery such as clathrin and the β2-adaptin subunit of the adaptor protein complex-2 (AP-2). In this way, β-arrestins couple GPCRs to clathrin-coated pits (CCPs) to facility receptor internalization (**Figure 6**). However, β-arrestin-independent internalization has also been described for some GPCRs [e.g., the protease-activated receptor 1 (PAR1; Paing et al., 2002) and the leukotriene B_4_ receptor BLT1 (Chen et al., 2004)] and might involve caveolae (lipid microdomains in the plasma membrane containing caveolin proteins that act as endocytic vehicles; Allen et al., 2007) or the direct interaction of GPCRs with endocytic proteins (e.g., AP-2; van Koppen and Jakobs, 2004). Desensitization and internalization are important feedback mechanisms that protect cells from overstimulation and malfunction of the desensitization machinery leads to various diseases. For example, almost all WHIM patients carry a mutation in CXCR4 that results in a premature stop codon. This causes the truncation of the C-terminus and results in impaired desensitization and internalization of CXCR4, leading to aberrant CXCR4 signaling (Balabanian et al., 2005; Kawai and Malech, 2009).

Besides S/T residues, other determinants in the C-terminus of GPCR can regulate internalization and trafficking. For example, AP-2 can directly bind to PAR1 by recognizing the YXXØ motif (Y is tyrosine, X is any amino acid, Ø is an amino acid with a bulky hydrophobic side chain). PAR1 internalizes independently of β-arrestin, but depletion of AP-2 by siRNA indeed inhibits constitutive internalization of this GPCR (Paing et al., 2006; Chen et al., 2011). AP-2 is also reported to bind to the C-terminus of the α_1b_-adrenergic receptor (α_1b_-AR) by recognizing a poly-arginine motif. Deletion of this motif inhibits α_1b_-AR internalization (Diviani et al., 2003). Furthermore, AP-2 can recognize di-leucine (LL or LI) motifs to induce the internalization of GPCRs such as CXCR2 (Fan et al., 2001), CXCR4 (Orsini et al., 1999), and β_2_AR (Gabilondo et al., 1997).

Internalized GPCRs traffic to endosomes where they are dephosphorylated by phosphatases (**Figure 6**). As a consequence, they recycle back to the cell surface to participate again in signaling. Alternatively, GPCRs can be sorted to lysosomes for degradation (Magalhaes et al., 2012). Although determinants for GPCR sorting are not completely understood, β-arrestins may regulate the fate of internalized GPCRs. GPCRs that transiently interact with β-arrestins recycle to the cell surface. In contrast, GPCRs that form stable complexes with β-arrestin are degraded. Factors that contribute to the stability of the GPCR/β-arrestin interaction include the presence of phosphorylated S/T clusters in the C-terminus of GPCRs (Oakley et al., 2001; Luttrell and Lefkowitz, 2002) and ubiquitination of β-arrestin (Shenoy and Lefkowitz, 2003). Different recycling sequences have been identified in the C-terminus of a considerable number of GPCRs that interact with recycling sorting proteins such as Na^+^/H^+^ exchanger regulatory factor 1 (NHERF) and *N*-ethylmaleimide-sensitive factor (NSF; Magalhaes et al., 2012). Alternatively, ubiquitination of GPCRs allows for recognition by the endosomal-sorting complex required for transport (ESCRT) machinery and targeting for degradation (Marchese and Trejo, 2013). However, ubiquitin- and ESCRT-independent mechanisms also contribute to target GPCR to lysosomes. The family of GPCR-associated sorting proteins (GASP) and sorting nexin-1 (SNX1) regulate the degradation of several GPCRs, but the exact mechanism is unknown (Marchese et al., 2008). On the other hand, GASP and SNX1 also bind to GPCRs that efficiently recycle after internalization (Hanyaloglu and von Zastrow, 2008; Marchese et al., 2008).

Importantly, β-arrestins not only arrest G protein-dependent signaling, but can also initiate signaling by serving as ligand-regulated scaffolds that recruit signaling proteins (Luttrell and Gesty-Palmer, 2010; **Figure 6**). For example, several MAP kinases (e.g., ERK1/2, JNK3, and p38) can be activated in a β-arrestin-dependent manner (DeWire et al., 2007). β-arrestin-dependent signaling has been implicated in the cardiovascular system, the immune system, and metabolic regulation, but also in pathological conditions such as cardiac failure and cancer (Luttrell and Gesty-Palmer, 2010).

#### Desensitization and trafficking of ORF74

**FIGURE 7:**
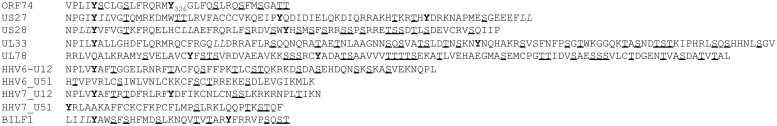
**Sequences of the C-terminus of the different HHV-encoded vGPCRs.** Sequences start at the conserved NPxxY motif. The start of the C-terminus of UL78 and BILF1, which lack the NPxxY motif, have been determined by sequence alignment with the other vGPCRs. S/T residues are underlined, Y residues are bold, and di-leucine motifs are italic. With the exception of UL78, all HCMV-encoded vGPCRs contain a di-leucine motif in their C-terminus. Although the C-terminus of the different vGPCRs differ in length, all receptors contain serine (S)/threonine (T) residues in their C-terminus. Only UL78 contains multiple S/T clusters (three or more S/T residues in a row). Most vGPCRs contain at least one Y residue. For some receptors, this Y residue is part of the NPxxY motif and unlikely to directly interact with proteins such as AP-2 as the NPxxY motif is located in TM7.

Whereas several studies have focused on cellular signaling via G proteins, details about signal termination and trafficking of ORF74 remain largely unknown. Examination of the C-terminus of ORF74 (**Figure 7**) reveals the presence of multiple S and T residues. Overexpression of GRK4, GRK5, and GRK6 indeed desensitize ORF74-induced PLC activation, cell proliferation, and foci formation (Bais et al., 1998; Geras-Raaka et al., 1998a). Although this suggests that S/T phosphorylation of ORF74 is involved in desensitization, direct evidence of ORF74 phosphorylation and subsequent β-arrestin recruitment is lacking. Surprisingly, overexpression of GRK2 does not reduce PLC activation by ORF74 (Bais et al., 1998; Geras-Raaka et al., 1998a). In contrast to some human chemokine receptors [e.g., CXCR2, CXCR4, and CCR5 (Neel et al., 2005)], the C-terminus of ORF74 lacks a di-leucine internalization motif. However, a classical tyrosine-based AP-2 binding motif (Y_326_GLF) is present in the C-terminus of ORF74. Indeed, ORF74 constitutively interacts with components from clathrin-coated vesicles, including AP-2, and siRNA-mediated knockdown of these components lead to increased expression of ORF74 at the cell membrane (Azzi et al., 2013). Furthermore, alanine-substitution of the Y residue within this AP-2 binding motif (ORF74-Y_326_A) inhibits the interaction with AP-2. ORF74-Y_326_A accumulates at the cell surface and barely in intracellular vesicles as compared to WT-ORF74 (Azzi et al., 2013). This suggests that Y_326_ is essential for the constitutive internalization of ORF74, but does not exclude a putative role for β-arrestin in ORF74 trafficking. Interestingly, ORF74-Y_326_A fails to downregulate Toll like receptor 4 (TLR4; Azzi and Gavard, 2014), which is a key player in the innate immune response against KSHV (Lagos et al., 2008). This indicates that ORF74 regulates the cell surface expression of immune proteins by constitutive internalization and provides a first clue of the relevance of ORF74 internalization. Whether TLR4 co-internalizes with ORF74 within a protein complex or whether ORF74 affects TLR4 expression via an autocrine/paracrine mechanism is hitherto unknown. Although ORF74 was shown to interact with the lysosome sorting adaptor GASP (Heydorn et al., 2004), no information is available about the fate of internalized ORF74. Furthermore, β-arrestin-dependent signaling of ORF74 has hitherto not been reported.

#### Desensitization and trafficking of BILF1

To our best knowledge, internalization and endocytic trafficking of BILF1 has not been reported. However, a C-terminus deletion mutant of BILF1 fails to downregulate MHC class I proteins (see also “EBV”; Griffin et al., 2013), indicating that the interaction between BILF1 and MHC class I proteins might result in co-internalization. The C-terminus of BILF1 contains several S and T residues and a single Y residue (**Figure 7**) that might act as β-arrestin and/or AP-2 binding sites, respectively.

#### Desensitization and trafficking of HCMV-encoded GPCRs

The C-terminus of US28 contains several S/T residues (**Figure 7**) and US28 is constitutively phosphorylated by GRK2, GRK5, PKC, and CK2 (Mokros et al., 2002; Miller et al., 2003). Alanine-substitution of these C-terminal S/T residues (US28-ST/A; Mokros et al., 2002) or deleting the C-terminus (US28(1-314); Miller et al., 2003; Waldhoer et al., 2003; Stropes et al., 2009) abrogated US28 phosphorylation and internalization, resulting in increased cell surface expression and constitutive signaling as compared to WT-US28 in heterologous expression systems (Miller et al., 2003; Waldhoer et al., 2003) and in cells infected with HCMV-US28(1-314; Stropes et al., 2009). US28, but not a phosphorylation-deficient mutant US28-S1-12A, induces translocation of β-arrestin2-GFP to the plasma membrane. However, β-arrestin2 resides in endocytic vesicles that are spatially distinct from the US28-expressing vesicles (Droese et al., 2004). Moreover, US28 internalization is unaffected in embryonic fibroblasts from β-arrestin knockout mice (Fraile-Ramos et al., 2003) and by a dominant-negative β-arrestin mutant (Droese et al., 2004). This indicates that US28 internalizes independently of β-arrestins. The C-terminus of US28 further contains a di-leucine motif and a single tyrosine residue within an YHSM-sequence. US28 co-localizes with AP-2 in intracellular vesicles (Droese et al., 2004) and siRNA-mediated knockdown of AP-2 inhibited US28 internalization (Fraile-Ramos et al., 2003). Alanine-substitution of the tyrosine residue in the C-terminus does not affect US28 internalization, but mutation of the di-leucine motif to alanine reduces the rapid internalization of US28 (Droese et al., 2004). However, a direct interaction between US28 and AP-2 has not been reported. US28 is primary located on early endosomes and recycling endosomes and recycles back to the plasma membrane after internalization (Fraile-Ramos et al., 2001). However, US28 also colocalizes with markers of lysosomes (Tschische et al., 2010) and interacts with SNX1 and GASP (Heydorn et al., 2004). US28 does not colocalize with lysosome markers when co-expressed with a dominant-negative mutant of GASP or siRNA targeting GASP (Tschische et al., 2010). Surprisingly, overexpression of GASP increases US28-mediated PLC activation, whereas shRNA-mediated knockdown of GASP or co-expression of a dominant-negative GASP mutant inhibits US28-mediated PLC activation (Tschische et al., 2010). These results indicate that GASP is involved in the regulation of US28 signaling. It is unknown whether GASP targets US28 to a particular cellular compartment to facilitate US28 signaling, whether GASP stabilizes a more active conformation of US28 or whether GASP acts as a signaling partner of US28.

Also UL33 (Fraile-Ramos et al., 2002), UL78 (Wagner et al., 2012), and US27 (Fraile-Ramos et al., 2002; Niemann et al., 2014) are primarily localized in endosomes of transfected or HCMV-infected cells and show constitutive internalization (Fraile-Ramos et al., 2002; Wagner et al., 2012). Different truncation mutants show that the distal end of the C-terminus of US27 (at least the last 14 residues) regulates its intracellular localization (Stapleton et al., 2012). This region contains a single S residue and a di-leucine motif (**Figure 7**), which might act as determinants for US27 internalization.

#### Desensitization and trafficking of Roseoloviruses-encoded GPCRs

HHV6-U51 internalizes in response to CCL2, CCL11, CCL19, and XCL1, as was shown by decreased cell surface expression of HHV6-U51 in a stably transfected erythroleukemia cells as quantified by flow cytometry (Catusse et al., 2008). However, the mechanism remains to be elucidated. Internalization and endocytic trafficking of the other three *Roseoloviruses*-encoded vGPCRs has hitherto not been studied. The C-terminus of HHV6-U12, HHV6-U51, HHV7-U12, and HHV7-U51 all contain multiple S and T residues (**Figure 7**), which might act as putative β-arrestin binding sites. In addition, HHV7-U12 contains a tyrosine residue in its C-terminus, which might facilitate AP-2 binding.

### MODULATION OF HUMAN GPCRs BY VIRAL GPCRs

**FIGURE 8:**
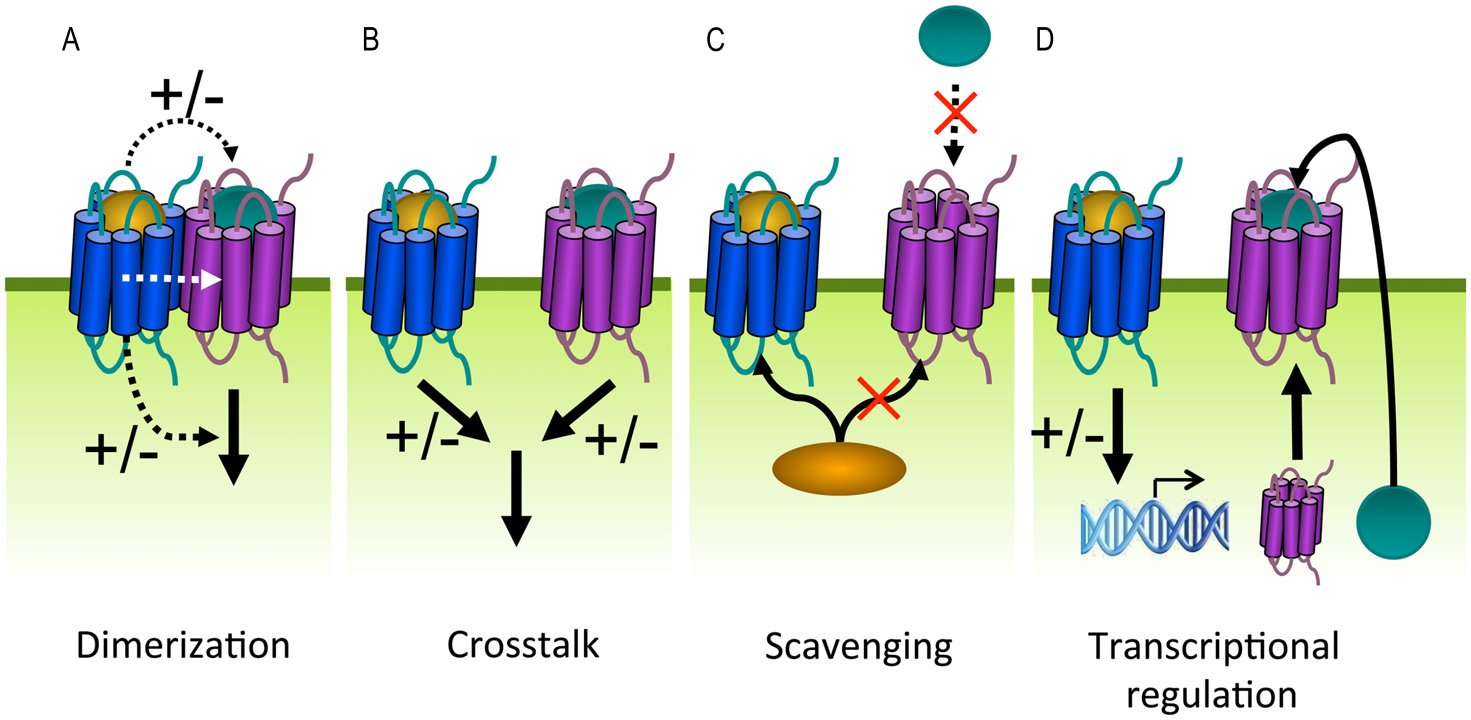
**G protein-coupled receptors can modulate each other’s function via different mechanisms.** **(A)** GPCRs can positively (+) or negatively (–) modulate (dashed black arrows) ligand binding, signaling (solid black arrow), or trafficking via allosteric interactions (dashed white arrow) within a heterodimer. **(B)** Crosstalk can be the result of signals (solid black arrows) that integrate downstream of two GPCRs. **(C)** Scavenging of a limited pool of shared signaling or scaffolding proteins (curved black arrow) might modulate signaling or ligand binding (dashed black arrow) of co-expressed GPCRs. **(D)** GPCRs can regulate the expression levels of other GPCRs, their ligands or signaling proteins.

Cells usually express multiple GPCR subtypes that do not function in isolation but generate integrated responses by modulating each other in dimers or via downstream crosstalk. GPCR heterodimerization can alter different aspects in the GPCR life cycle including ligand binding, signaling and trafficking (**Figure 8A**). For example, both positive and negative binding cooperativity has been observed within GPCR heterodimers as a consequence of intermolecular allosteric interactions. This explains why the ligand of one receptor is able to displace the ligand of a co-expressed receptor. Negative binding cooperativity has been shown for the chemokine receptors CCR2, CCR5, and CXCR4 (El-Asmar et al., 2005; Sohy et al., 2007, 2009). Furthermore, GPCR heterodimerization may lead to potentiation or attenuation of signaling or even changes in G protein selectivity. This was shown for the dopamine D_1_ and D_2_ receptors that generate a novel PLC-mediated Ca^2+^ signal when co-expressed (Lee et al., 2004). The obligatory dimerization between the GABA_B1_ and GABA_B2_ receptors forms one of the best examples showing the functional relevance of dimerization with regard to proper cell surface delivery. When expressed on their own, the two subunits are non-functional. GABA_B1_ is unable to leave the ER after synthesis because this receptor contains an ER retention motif within its C-terminus. The GABA_B2_ receptor lacks this motif and traffics to the cell surface but is unable to bind ligands. When co-expressed, the two receptors physically assemble via a coiled-coil interaction of their C-terminuses and masking the ER retention motif of GABA_B1_ (Margeta-Mitrovic et al., 2000; Pagano et al., 2001). Heterodimerization might also promote co-internalization of both receptors after stimulation of only one protomer. Conversely, the internalization of one receptor can be inhibited by forming heterodimers with a receptor that is resistant to agonist-induced internalization (Terrillon and Bouvier, 2004).

On the other hand, GPCRs can modulate each other’s function without direct association. Two GPCRs might integrate their signals downstream of receptor activation when they share signaling molecules (Prezeau et al., 2010; **Figure 8B**). For example, the inhibitory and activating signals of Gα_i_ and Gα_s_-coupled receptors converge at the level of AC, resulting in balanced cAMP levels. Dimerization and downstream crosstalk are often difficult to distinguish (Vischer et al., 2011). Alternatively, GPCRs might influence ligand binding or signaling of other GPCRs by scavenging shared signaling proteins or depleting signaling molecules from a limited pool (**Figure 8C**). For example, as G protein and/or β-arrestin coupling are required for high affinity agonist binding to some GPCRs, one GPCR might impair ligand binding of a second GPCR by depleting the available G protein or β-arrestin pools (Chabre et al., 2009). Finally, GPCRs might modulate ligand or receptor expression levels by regulating transcription/translation (**Figure 8D**). Viral and human GPCRs may modulate each other to alter the functional properties of the latter in favor of the virus.

#### Modulation of human GPCRs by ORF74

Examples of modulating human GPCRs by ORF74 are rare and fairly understudied. One study shows that the co-expression of ORF74 inhibits Ca^2+^ mobilization induced by the thyrotropin-releasing hormone receptor and the muscarinic acetylcholine M_1_ receptor in oocytes, HEK293 EM cells, and mouse pituitary AtT20 cells (Lupu-Meiri et al., 2001). This effect was further enhanced by CXCL1 and inhibited by CXCL10. Depletion of intracellular Ca^2+^ pools as a result of the constitutive signaling of ORF74 was proposed as the underlying mechanism (Lupu-Meiri et al., 2001).

#### Modulation of human chemokine receptors by BILF1

BILF1 physically interacts with several chemokine receptors from the CCR and CXCR family (Vischer et al., 2008) and the heteromeric complex between BILF1 and CXCR4 is composed of at least four GPCRs (Nijmeijer et al., 2010). Although BILF1 does not bind to CXCL12 (or other human chemokines, see Section “Chemokine Binding to EBV-Encoded BILF1”), co-expression of BILF1 inhibits CXCL12 binding to human CXCR4 and consequently inhibits CXCR4 signaling (Nijmeijer et al., 2010). Overexpression of Gα_i_ restores CXCL12 binding and signaling through CXCR4, indicating that the impaired CXCL12 binding to CXCR4 is the consequence of constitutive Gα_i_ scavenging by BILF1 rather than the transmission of conformational changes across the BILF1/CXCR4 heteromeric complex (Nijmeijer et al., 2010).

#### Modulation of human GPCRs by HCMV-encoded GPCRs

The human chemokine receptor CCR1 binds with high affinity to CCL5, but only induces a small PTX-sensitive activation of NF-κB. However, when US28 is co-expressed, CCL5 induces a robust PTX-sensitive increase in NF-κB activation (Bakker et al., 2004), which is mediated by CCR1 as co-expression of chemokine binding-deficient mutant ΔN-US28 (Casarosa et al., 2003b) also enables NF-κB signaling in response to CCL5 (Bakker et al., 2004). On the other hand, US28-R^3.50(129)^A fails to unmask CCR1 signaling, showing that the crosstalk between US28 and CCR1 requires the constitutive activity of US28 (Bakker et al., 2004). Likewise, US28 and ΔN-US28 (but not US28-R^3.50(129)^A) potentiates LPA-mediated Ca^2+^ mobilization in HCMV-infected smooth muscle cells (Miller et al., 2012).

Interestingly, while US27 and UL78 are described as silent orphan receptors, they are able to modify signaling of CXCR4. US27 increases CXCR4 expression in HEK293T cells and potentiates Ca^2+^ mobilization and chemotaxis in response to CXCL12 (Arnolds et al., 2013), whereas UL78 inhibits these CXCR4-mediated responses in monocytes (Tadagaki et al., 2012). These opposite effects might reflect the need of the virus to escape immune surveillance or promote viral spread by cell migration during different stages of HCMV infection.

UL33 and UL78 form heterodimers with human CCR5 and the functional consequences of these interactions depend on the dimerization partner and functional read-out (Tadagaki et al., 2012). Both UL33 and UL78 impair CCL5-induced internalization of CCR5. However, while UL33 almost completely blocks CCR5-induced PLC activation and Ca^2+^ mobilization, UL78 increases these responses. On the other hand, both viral GPCRs had a negative effect on CCR5-mediated cell migration (Tadagaki et al., 2012).

#### Modulation of human GPCRs by Roseoloviruses-encoded GPCRs

Comparable to the crosstalk between US28 and CCR1, HHV7-U12 and U51 unmask CCL19 and CCL22-induced Ca^2+^ mobilization mediated by CCR4 and CCR7 in murine L1.2 cells (Tadagaki et al., 2007). In the absence of U12 and U51, CCR4 only responds to CCL22 and CCR7 only to CCL19 (**Figure 4**). Furthermore, although CCL19 and CCL22 induce U12- and U51-mediated Ca^2+^ mobilization in human erythroleukemia K562 cells (Tadagaki et al., 2005), these vGPCRs are unable to promote Ca^2+^ mobilization in response to CCL19 or CCL22 in murine L1.2 cells (Tadagaki et al., 2007). Surprisingly, these unmasked signals are not observed in cell migration assays (Tadagaki et al., 2007).

### MODULATION OF HUMAN RTKs BY VIRAL GPCRs

**FIGURE 9:**
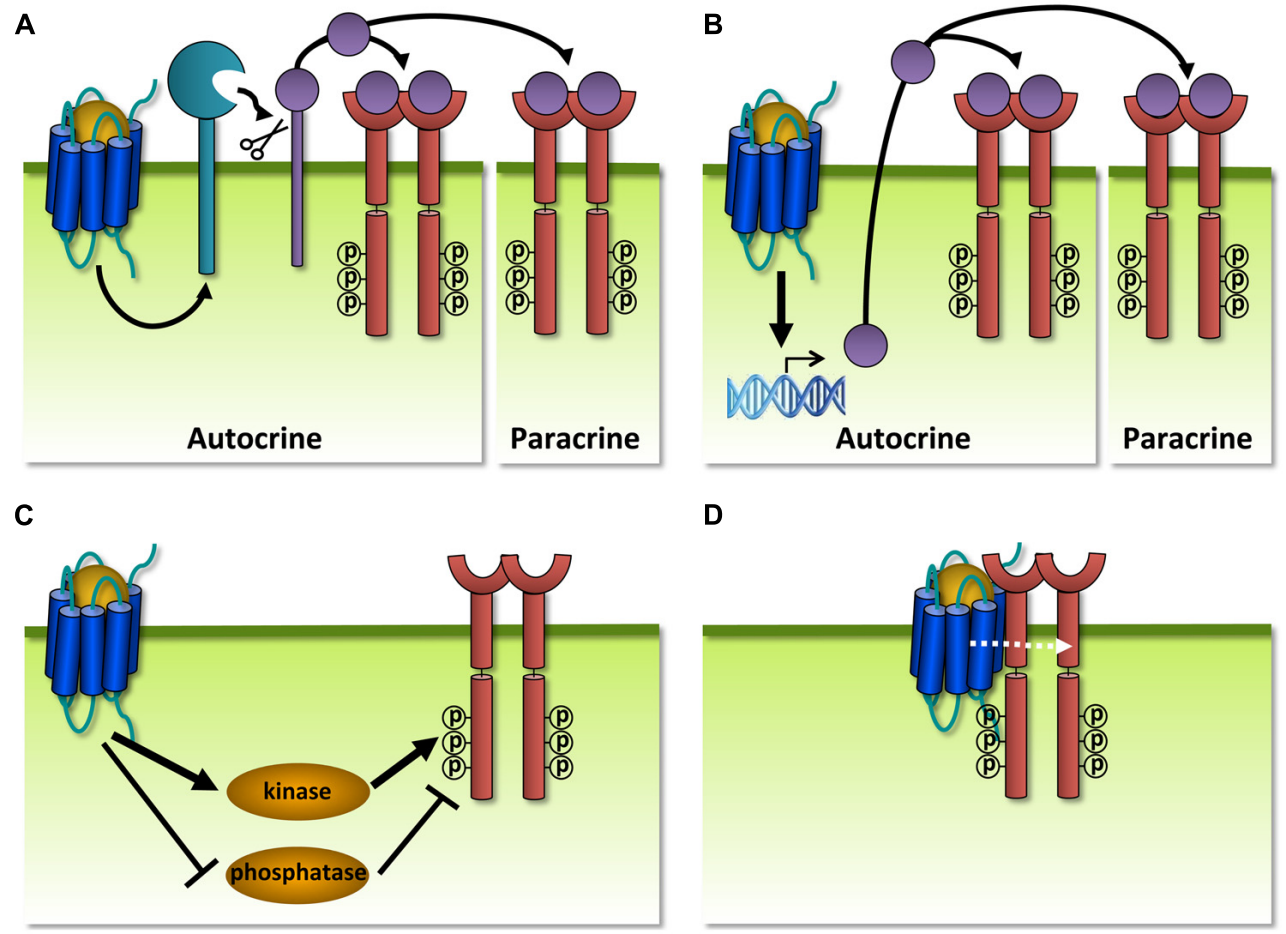
**Different mechanisms of receptor tyrosine kinase (RTK) transactivation.** **(A)** GPCR-induced activation of a membrane-anchored metalloproteinase results in the release of a growth factor which activates its cognate RTK in a autocrine and/or paracrine manner. **(B)** GPCRs regulate the expression and secretion of growth factors that transactivate RTKs in a autocrine and/or paracrine manner. **(C)** Ligand-independent transactivation of RTKs via the GPCR-induced activation (black arrow) or inhibition of tyrosine kinases or phosphatases, respectively. **(D)** GPCRs transactivate RTKs within a protein complex, possibly via allosteric interactions (dashed white arrow).

**FIGURE 10:**
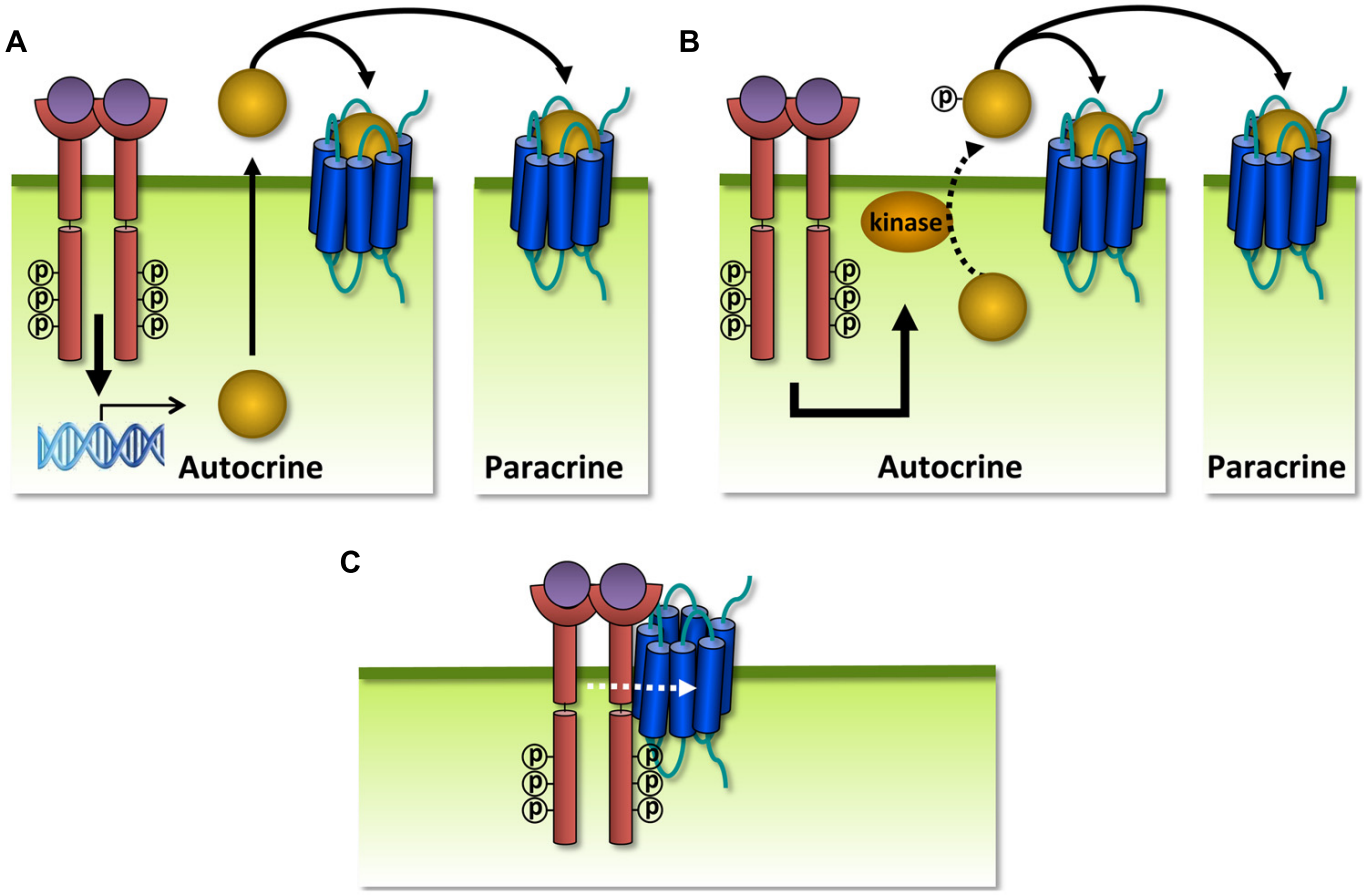
**Different mechanisms of GPCR transactivation.** **(A)** RTK-induced *de novo* synthesis of GPCR ligands that activate their cognate receptor in an autocrine and/or paracrine manner. **(B)** RTK-induced enzymatic activation and secretion of GPCR ligands that activate their cognate receptor in an autocrine and/or paracrine manner. **(C)** RTKs might transactivate interacting GPCRs from internalized vesicles, leading to extracellular-signal-regulated kinases (ERK) activation. The exact mechanism and requirement of the RTK/GPCR interaction for transactivation is not clear.

In addition to GPCRs that modulate each other’s functioning, also GPCRs and RTKs are organized within communication networks. RTKs comprise a class of transmembrane proteins that are structurally and functionally distinct from GPCRs. RTKs are commonly activated by growth factors that induce formation of receptor dimers, resulting in the autophosphorylation of intracellular tyrosine residues and the subsequent binding of adaptor proteins that activate downstream signaling pathways such as MAP kinases (Lemmon and Schlessinger, 2010). The term ‘transactivation’ is often used to describe RTK activation by a GPCR ligand without the addition of growth factors or vice versa (Daub et al., 1996). GPCRs and RTKs can transactivate each other via different mechanisms, with GPCRs acting either upstream (**Figure 9**) or downstream (**Figure 10**) of RTKs. RTK transactivation can be ligand-dependent resulting in both autocrine and/or paracrine signaling (**Figures 9A,B**) or ligand-independent (**Figures 9C,D**; Delcourt et al., 2007a). Only ligand-dependent mechanisms can lead to the transactivation of RTKs on neighboring cells that do not co-express the GPCR. One of the best characterized ligand-dependent mechanisms for RTK transactivation involves the GPCR-induced activation of a membrane-anchored metalloproteinase, resulting in the release of an membrane-anchored growth factor-precursor which subsequently activates its cognate receptor (**Figure 9A**; Wetzker and Bohmer, 2003). This mechanism has only been described for the transactivation of the epidermal growth factor receptor (EGFR) and in a single case for the insulin-like growth factor 1 receptor (IGF-1R; Oligny-Longpre et al., 2012). Alternatively, transactivation of RTKs might also involve GPCR-induced *de novo* synthesis of growth factors (**Figure 9B**). RTK transactivation by ligand-independent mechanisms involves the phosphorylation of the RTK by a tyrosine kinase acting downstream of GPCR signaling (**Figure 9C**), GPCR-induced inactivation of tyrosine phosphatases that control RTK activity (**Figure 9C**; Wetzker and Bohmer, 2003) or the formation of a GPCR/RTK-signaling complex (**Figure 9D**; Delcourt et al., 2007a). Transactivation of RTKs often accounts for the proliferation, differentiation, migration, and survival responses promoted by GPCRs. For example, transactivation of IGF-1R by GABA_B_ receptor protects neurons from apoptosis (Tu et al., 2010). An antibody that prevents IGF-1 binding to IGF-1R could not antagonize this transactivation. Instead, the Gα_i/o_-inhibitor PTX, a PLC inhibitor, a Ca^2+^ chelator, and siRNA-mediated knockdown of FAK1 all impair IGF-1R transactivation by GABA_B_ and show the requirement of these downstream proteins and second messenger. Furthermore, IGF-1R was co-immunoprecipitated with GABA_B1_, but there is no evidence that an interaction between the two receptors is essential for the observed crosstalk (Tu et al., 2010).

Reciprocally, examples of RTKs that transactivate GPCRs are less abundant and involve the *de novo* synthesis (**Figure 10A**) or the enzymatic activation (**Figure 10B**) of GPCR ligands that act in an autocrine/paracrine manner, or a physical interaction with GPCRs (**Figure 10C**). For instance, activation of IGF-1R results in the upregulation of CCL5 on the transcriptional level and the subsequent activation of CCR5-mediated chemotaxis (**Figure 10A**; Mira et al., 2001). Furthermore, transactivation of the sphinogosine 1 phosphate receptor S1P1 is regulated by the formation of its ligand S1P from a precursor via IGF-1R-, TrkA-, or plateled-derived growth factor receptor (PDGFR)-mediated activation of sphingosine kinase (SphK; **Figure 10B**; Hobson et al., 2001; Toman et al., 2004; El-Shewy et al., 2006). Ligand-independent mechanisms also exist for transactivation of GPCRs and involve the formation of constitutive complexes between CXCR4 and IGF-1R (Akekawatchai et al., 2005), pituitary AC activating polypeptide type 1 receptor (PAC1R) and IGF-1R (Delcourt et al., 2007b) and between S1P1 and PDGFR (Alderton et al., 2001). However, the nature and the role of these interactions for GPCR transactivation are not well understood but might involve the signaling from intracellular vesicles of co-internalized GPCR/RTK complexes (**Figure 10C**; Waters et al., 2003).

#### Modulation of human RTKs by ORF74

Studies examining the modulation of human RTKs by ORF74 have hitherto been limited to the angiogenic VEGF receptors VEGFR-1 and VEGFR-2 and the mechanism depicted in **Figure 9B**. VEGF plays an important role in the angiogenesis of KS and a small molecule inhibitor of the VEGFR-1 has a positive outcome in the majority of AIDS-related KS patients in a phase I study (Arasteh and Hannah, 2000). ORF74-expressing endothelial cells increase VEGF secretion (Bais et al., 1998) via p38- and MAP kinase-mediated activation of HIF-1α (Sodhi et al., 2000), leading to VEGFR-2 activation and the subsequent survival (Bais et al., 2003), growth, and microtubule formation of endothelial cells (Bais et al., 1998). These effects could be inhibited by VEGF (Bais et al., 1998) or VEGFR-2 blocking antibodies (Bais et al., 2003). Indeed, *in vivo* models show that transgenic expression of ORF74 in mice results in VEGF secretion and the development of highly vascularized lesions (Yang et al., 2000).

Besides the angiogenic role of VEGF/VEGFR in KS, IGF-1R (Catrina et al., 2005), and PDGFR (Rossi et al., 2009) also play a role in KS. Furthermore, studying the KSHV secretome reveals secretion of several growth factors, including IGF-1, PDGF and EGF (Schwarz and Murphy, 2001; Jensen et al., 2005; Sharma-Walia et al., 2010). However, whether ORF74 transactivates human RTKs other than the VEGF receptors remains to be elucidated.

#### Modulation of human RTKs by other vGPCRs

Co-transfection of US28 and the membrane-anchored precursor Heparin-binding EGF-like growth factor (HB-EGF) results in increased levels of soluble EGF in the supernatant of intestinal epithelial Caco-2 cells as compared to US28-deficient Caco-2 cells, suggesting that US28 cleaves HB-EGF (Bongers et al., 2012). Furthermore, transgenic co-expression of US28 and HB-EGF in the intestines of mice results in an increased incidence and faster development of polyps as compared to mice only expressing HB-EGF (Bongers et al., 2012). These results indicate that US28 might transactivate EGFR.

Moreover, US28 constitutively promotes VEGF secretion (Maussang et al., 2006) via COX-2 (Maussang et al., 2009b) and STAT3 (Slinger et al., 2010) in transfected NIH-3T3 cells and HCMV-infected glioblastoma cells (Maussang et al., 2006; Soroceanu et al., 2011). Furthermore, *in vivo* studies show that US28 increases the VEGF plasma levels in a xenograft mouse model that developed highly vascularized tumors (Maussang et al., 2006), indicating that US28 contributes to an angiogenic phenotype in proliferative diseases.

Constitutive BILF1 signaling also results in VEGF secretion (Lyngaa et al., 2010), but knowledge about a mechanism and any downstream effects is lacking.

Besides a role in transactivation, EGFR (Wang et al., 2003), and PDGFR (Soroceanu et al., 2008) have been proposed to act as co-receptors for HCMV entry. HCMV directly interacts with EGFR or PDGFR via its envelop glycoprotein gB. Antibodies blocking EGFR or PDGFR, or siRNA-mediated knockdown of PDGFR inhibit HCMV gene expression and viral replication. EGFR- or PDGFR-negative cells are not permissive to HCMV infection, but expression of EGFR or PDGFR renders these cells susceptible to HCMV. However, others were unable to reproduce the results with the PDGFR blocking antibodies and silencing PDGFR with shRNA did not inhibit HCMV entry. However, overexpression of PDGFR enhanced HCMV entry (Vanarsdall et al., 2012). It was proposed that PDGFR does not interact directly with HCMV but enhances HCMV entry via a non-canonical pathway involving dynamin-dependent endocytosis (Vanarsdall et al., 2012). Likewise, the role of EGFR as a HCMV co-receptor has been challenged by contradicting results showing that an EGFR blocking antibody or small molecule inhibitor did not decrease HCMV entry in fibroblast, epithelial or endothelial cell (Isaacson et al., 2007). These contradicting results might be due to cell type-dependent mechanisms underlying HMCV entry and other receptors might substitute for EGFR/PDGFR. This could explain why EGFR is not expressed on all HCMV-permissive cell types, such as monocytes/macrophages, dendritic cells, and neutrophils (Isaacson et al., 2007).

## CONCLUDING REMARKS AND FUTURE PERSPECTIVES

**FIGURE 11:**
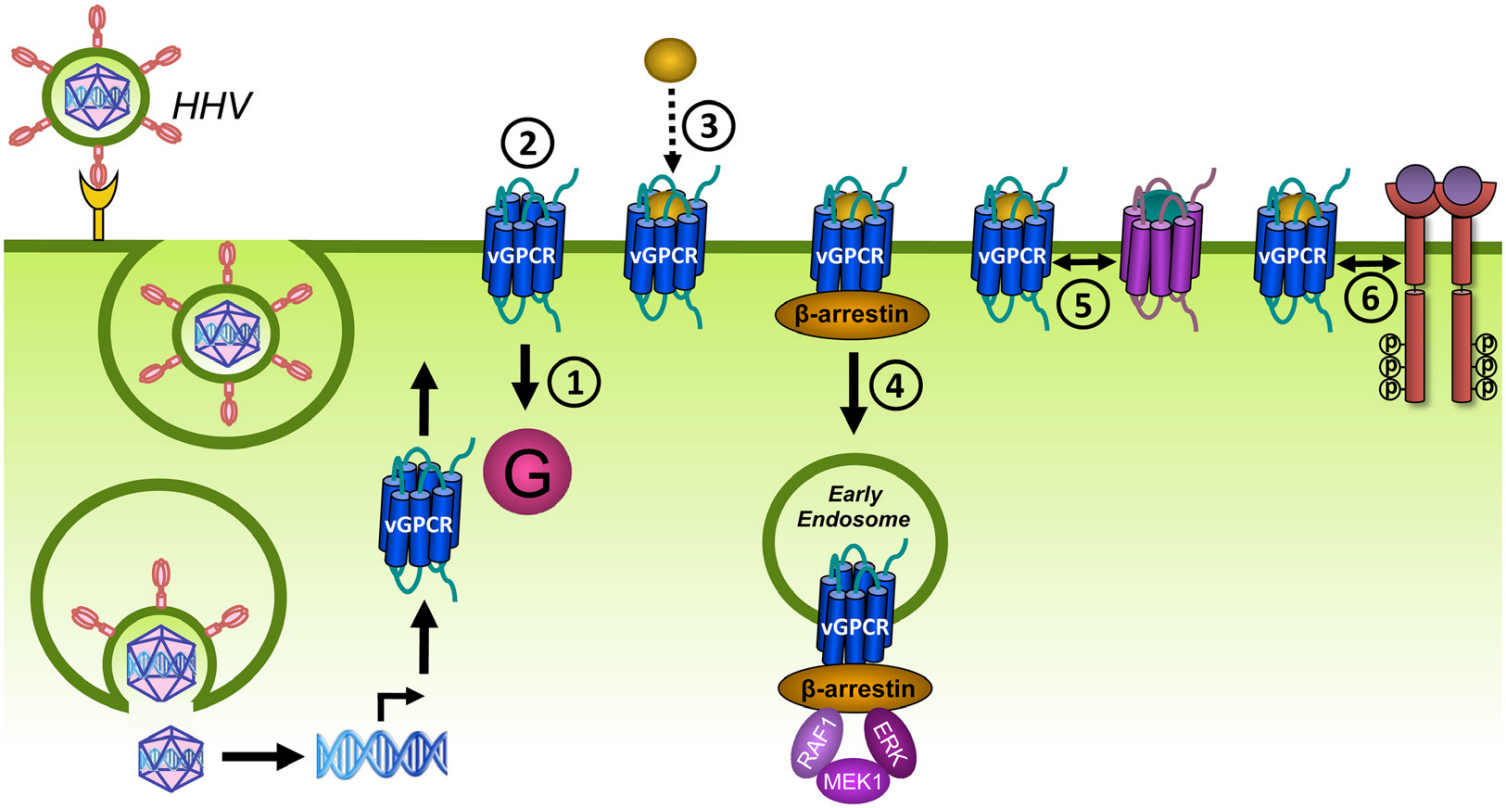
**Viral G protein-coupled receptors can modulate cellular signaling by means of different mechanisms.** vGPCR are expressed at the cell surface of HHV-infected cells. For most vGPCRs, canonical mechanisms of host cell modulation has been studied in detail and shows that vGPCRs can signal through G proteins **(1)** in a constitutively active manner **(2)**. Chemokine binding might modulate constitutive signaling **(3)**. Less knowledge is available on non-canonical mechanisms that involve the interaction of vGPCRs with proteins from the endocytic machinery (e.g., β-arrestin) **(4)**. In addition, vGPCR may modulate the function of human GPCRs **(5)** or RTKs **(6)**.

Human herpesviruses have successfully developed multiple strategies to escape immune surveillance and promote viral dissemination, which resulted in a high infection rate among the human population. Herpesviruses are usually harmless for most people, but can cause severe pathology in immunocompromised patients. All herpesviruses from the β and γ subfamily encode at least one vGPCR that shows homology to human chemokine receptors. These vGPCRs have been modified to obtain unique features, including constitutive activity and binding of a broad range of chemokines, and are used by the virus to take over the control of the host cell for its own benefit. In this review, we described six different ways by which vGPCRs (potentially) modulate cellular signaling (**Figure 11**). Constitutive and chemokine-induced G protein signaling have exhaustively been studied for most vGPCRs using *in vitro* heterologous expression and HCMV-infection models, as well as xenograft and transgenic *in vivo* models. Although vGPCRs have been detected in (patho)physiological patient samples (e.g., HCMV-positive glioblastoma tumors or KS lesions), little is yet known on their *in situ* (constitutive) signaling activities. Constitutive signaling is proportional to receptor expression levels, hence quantification of receptor levels in patient samples might allow some comparison with experimental models. Furthermore, cellular signaling pathways activated by vGPCRs might be cell type-dependent. For example, BILF1 activates NF-kB in COS-7 cells but not in Burkitt’s lymphoma cells or lymphoblastoid B cells (Beisser et al., 2005), showing the importance of using (patho)-physiologically relevant cell systems. Whereas many signaling properties of vGPCRs have been studied in recombinant systems using conventional cell lines (i.e., COS-7 cells), also disease-relevant cell lines such as glioblastoma cell lines for US28 and primary B cells from KS patients for ORF74 are used. Furthermore, HCMV-infected cells are often used to study US28 signaling in the viral context. HCMV-mutants such as US28-R^3.50(129)^A and ΔN-US28 have been developed to study G protein-coupling and chemokine binding in HCMV-infected cells instead of recombinant systems (Stropes and Miller, 2008). Such tools are useful to study the signaling of the other vGPCRs in a viral setting.

In this review we further discussed how vGPCRs exploit human proteins from the endocytic machinery or modulate the signaling of human GPCRs or RTKs (**Figure 11**). These subjects have mainly been neglected and are fairly understudied for most vGPCRs. Although endocytosis of US28 was the subject of several studies, still many questions remain unanswered. For example, S/T residues in the C-tail of US28 seem to be important for endocytosis (Mokros et al., 2002), yet the exact role of β-arrestin is still uncertain (Fraile-Ramos et al., 2003; Droese et al., 2004). Furthermore, the endocytic trafficking of most other vGPCRs have hitherto escaped attention. Are constitutively active vGPCRs internalized in the absence or presence of chemokines and if so, how is endocytic trafficking regulated? Does endocytosis contribute to viral dissemination by co-internalization of proteins key for anti-viral immune responses? Do US28 and BILF1, which possibly also contribute to tumor formation via G protein-independent signaling (see “G Protein-Dependent BILF1 Signaling” and “G Protein-Dependent Signaling of HCMV-Encoded GPCRs”) signal via β-arrestin-dependent mechanisms from intracellular compartments? Do apparent silent vGPCRs (e.g., UL78) signal via G protein-independent mechanisms and/or modulate GPCRs (chemokine receptors in particular) or RTKs through dimerization and/or transactivation? These subjects warrant further investigation to gain insight in the different properties of vGPCRs.

Since vGPCRs are identified to modify cellular signaling and are associated with HHV-associated pathologies, they might serve as potential drug targets. Specific inhibitors targeting vGPCR functioning might be used as research tools or for clinical antiviral intervention. Small non-peptidergic compounds that inhibit constitutive activity and chemokine binding have hitherto only been developed for US28. However, these US28 compounds display a low μM potency despite significant optimization efforts (Hulshof et al., 2005, 2006; Vischer et al., 2010; Kralj et al., 2011, 2013, 2014). Nanobodies are the antigen-binding fragments of a unique class of heavy chain-only antibodies found in camelids and are gaining popularity as targets for GPCRs due to their relative small size, high affinity, and specificity (Mujic-Delic et al., 2014). Nanobodies targeting CXCR4 and CXCR7 have recently been developed and induce CXCR4-mediated stem cell mobilization in cynomolgus monkeys, inhibit CXCR4-mediated HIV entry (Jahnichen et al., 2010) and inhibit CXCR7-mediated head and neck cancer tumor growth in a xenograft mouse model (Maussang et al., 2013), respectively. The nanobody-based targeting of vGPCRs might be an attractive and promising strategy for the development of research tools, diagnostics, and/or therapeutics.

Taken together, tools targeting viral GPCRs and knowledge on the mechanisms by which vGPCRs modulate cellular signaling will provide insight into viral spread and herpesvirus-associated pathologies.

## Conflict of Interest Statement

The authors declare that the research was conducted in the absence of any commercial or financial relationships that could be construed as a potential conflict of interest.
